# Reconstruction of the full-length transcriptome atlas using PacBio Iso-Seq provides insight into the alternative splicing in *Gossypium australe*

**DOI:** 10.1186/s12870-019-1968-7

**Published:** 2019-08-19

**Authors:** Shouli Feng, Min Xu, Fujie Liu, Changjiang Cui, Baoliang Zhou

**Affiliations:** State Key Laboratory of Crop Genetics & Germplasm Enhancement, MOE Hybrid Cotton R&D Engineering Research Center, Nanjing Agricultural University, Nanjing, 210095 Jiangsu People’s Republic of China

**Keywords:** Alternative splicing (AS), *Gossypium australe*, Pacific biosciences single-molecule long-read isoform sequencing (Iso-Seq), Transcriptome

## Abstract

**Background:**

*Gossypium australe* F. Mueller (2*n* = 2*x* = 26, G_2_ genome) possesses valuable characteristics. For example, the delayed gland morphogenesis trait causes cottonseed protein and oil to be edible while retaining resistance to biotic stress. However, the lack of gene sequences and their alternative splicing (AS) in *G. australe* remain unclear, hindering to explore species-specific biological morphogenesis.

**Results:**

Here, we report the first sequencing of the full-length transcriptome of the Australian wild cotton species, *G. australe,* using Pacific Biosciences single-molecule long-read isoform sequencing (Iso-Seq) from the pooled cDNA of ten tissues to identify transcript loci and splice isoforms. We reconstructed the *G. australe* full-length transcriptome and identified 25,246 genes, 86 pre-miRNAs and 1468 lncRNAs. Most genes (12,832, 50.83%) exhibited two or more isoforms, suggesting a high degree of transcriptome complexity in *G. australe*. A total of 31,448 AS events in five major types were found among the 9944 gene loci. Among these five major types, intron retention was the most frequent, accounting for 68.85% of AS events. 29,718 polyadenylation sites were detected from 14,536 genes, 7900 of which have alternative polyadenylation sites (APA). In addition, based on our AS events annotations, RNA-Seq short reads from germinating seeds showed that differential expression of these events occurred during seed germination. Ten AS events that were randomly selected were further confirmed by RT-PCR amplification in leaf and germinating seeds.

**Conclusions:**

The reconstructed gene sequences and their AS in *G. australe* would provide information for exploring beneficial characteristics in *G. australe*.

**Electronic supplementary material:**

The online version of this article (10.1186/s12870-019-1968-7) contains supplementary material, which is available to authorized users.

## Background

Cotton (*Gossypium* spp.) is not only the leading natural fibre resource but also the third largest field crop in terms of edible oil seed tonnage [1]. However, *Gossypium* species are characterized by the presence of small, darkly pigmented lysigenous glands that deposit gossypol, which is toxic to humans and monogastric animals [2, 3]. Therefore, cottonseed protein or oil containing gossypol is inedible. *G. australe* F. Mueller, a wild diploid cotton species (2*n* = 2*x* = 26, G_2_ genome), grows in a limited area of central and northern Australia. This species has many desirable characteristics, such as resistance to pests (aphids and mites) and diseases (Fusarium and Verticillium wilts) and tolerance to abiotic stresses (such as salinity, heat and drought). More importantly, it contains immature lysigenous glands but no terpenoid aldehydes, and its pigment glands appear only after seed germination; thus, little gossypol is deposited in the dormant seeds of the species [4, 5]. This distinguishing characteristic, called delayed gland morphogenesis, has the potential to enable the large-scale direct use of cottonseed for edible proteins and oils. Thus, *G. australe* could be a useful resource for the improvement of tetraploid cultivated species. However, *G. australe* still lacks a genome reference. To further understand the molecular genetic basis of delayed gland morphogenesis and exploit the stress tolerance of *G. australe* for application in breeding, it is necessary to obtain the nucleotide sequences of its genes.

A transcriptome provides a fast and economical method to systematically characterize gene models for one species without a genome reference. Recently, high-throughput RNA sequencing (RNA-Seq) via second-generation sequencing technologies has been widely used in eukaryotic transcriptome analyses [6]. However, short reads require very large computational assemblies and cannot span full-length transcripts, reducing the accuracy of gene model prediction [7, 8]. Full-length (FL) cDNA clone sequencing using Sanger sequencing was the first established method and has historically represented the gold standard for genome annotation projects [9]. However, this method has fallen out of fashion due to its low throughput and high cost. Recently, single-molecule long-read sequencing technology has been widely used in transcriptome sequencing because it provides particularly long reads with high throughput [8–10]. The use of Pacific Biosciences long-read isoform sequencing (Iso-Seq) thus offers a better alternative for sequencing more complete (i.e.*,* FL) transcriptomes, and this technique has been successfully used for predicting and validating gene models [8]. Considering its better ability to completely sequence both the 5′ and 3′ untranslated regions and the poly (A) tails of cDNA molecules, its higher accuracy for identifying alternative isoforms [10–12], alternative polyadenylation [12, 13] and its increased power to distinguish non-coding RNA (ncRNA) [12, 14], Iso-Seq is regarded as having an advantage over RNA-Seq for reconstructing the coding genome [15, 16].

In the present study, we applied Iso-Seq technology to generate a FL transcriptome for *G. australe*. To widen the coverage of tissue-specific transcript isoforms, we multiplexed ten tissues at different developmental stages (Additional file 1:Figure S1) and pooled them for subsequent sequencing. We applied the Iso-Seq protocol to analyse this FL transcriptome and reconstruct the *G. australe* transcript loci. *G. australe* Iso-Seq analysis uncovered complex alternative splicing (AS) in the gene transcript loci, extending our knowledge far beyond the existing resources in terms of breadth (genes coverage) and depth (transcripts per gene). Herein, we not only systematically characterize the complexity of the *G. australe* FL transcriptome but also provide a valuable resource for investigating the molecular genetic basis of delayed gland morphogenesis.

## Results

### *G. australe* FL transcriptome sequencing and bioinformatics pipeline

To identify as many transcripts as possible, we extracted high-quality RNA from ten tissues of *G. australe* F. Mueller at different developmental stages or treatments and verified that the RNA quality met the requirements for reverse transcription (Additional file 1:Figure S1). Then, we sequenced barcoded single-molecule real-time (SMRT) Bell libraries on the PacBio Sequel platform using the latest P6–C4 chemistry. As a result, 1,001,928 circular consensus sequences (CCS) were obtained from five SMRT cells, and the expected distribution of transcript lengths ranged from 200 to 10,000 nucleotides (Additional file 2:Figure S2). All CCS reads were further classified into 656,418 full-length non-chimeric (FLnc) reads, which had the 5′ and 3′ barcoded primers and the poly (A) tail simultaneously, and 242,894 non-full-length (nFL) sequences lacking any one of the 5′ primer, 3′ primer or poly (A) tail. After using the Iso-Seq cluster’s clustering algorithm, ICE (iterative clustering for error correction), we finally obtained 320,357 FL consensus isoform sequences (Additional file 14:Table S1). Afterward, we employed ‘LoRDEC’ [17] to correct errors using RNA-Seq reads (Additional file 15:Table S2). Thus, about 22 million (3.11%) errors were corrected, with 0.77% insertions, 1.75% deletions and 0.60% SNPs, being consistent with the above polished consensus isoforms (Additional file 15:Table S2).

### Reconstruction of *G. australe* transcript loci

**Fig. 1:**
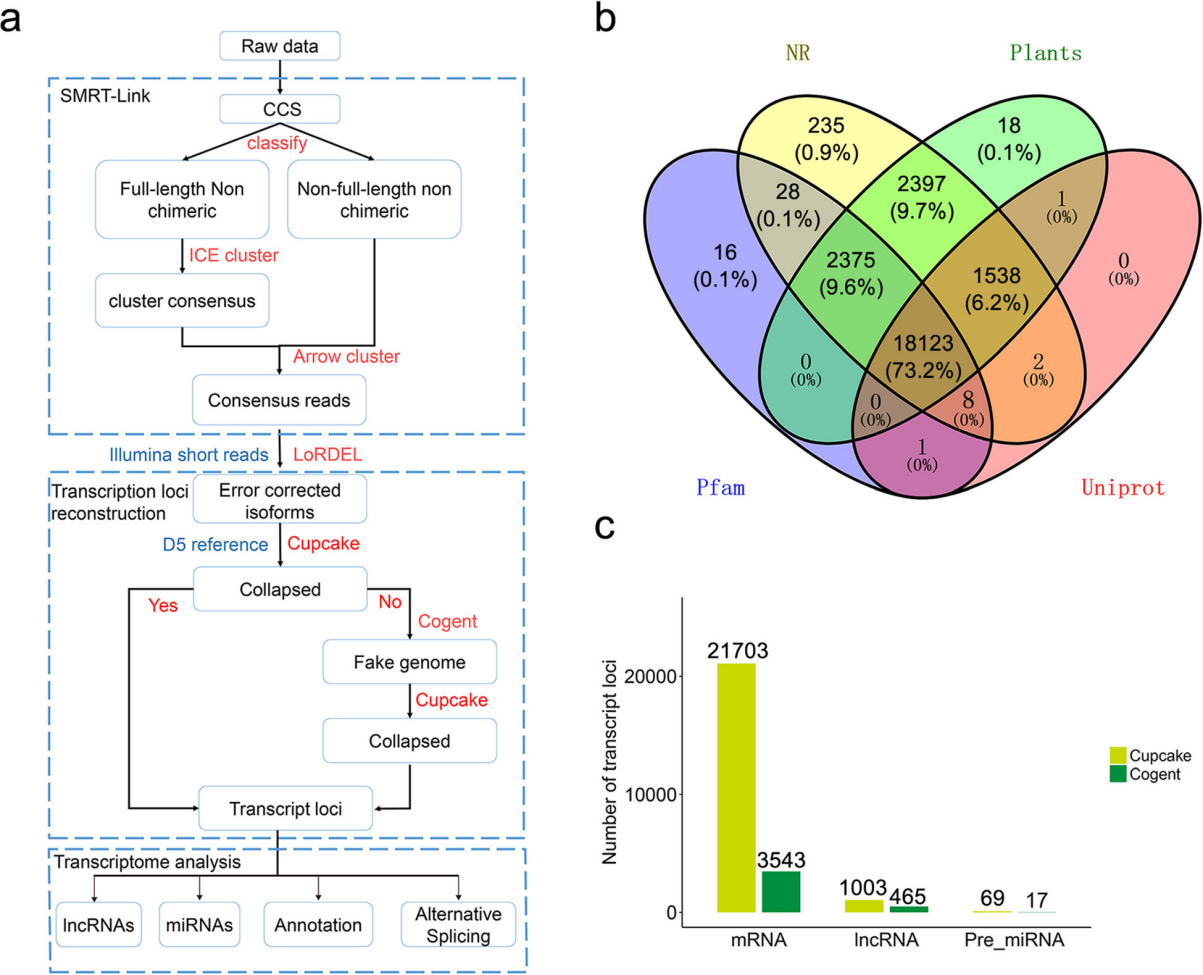
Reconstruction of *G. australe* FL transcript loci from Iso-Seq. **a** Pipeline used for reconstruction of FL transcript loci from Iso-Seq using a combination of the ‘has genome’ and ‘no genome’ strategies. **b** Functional annotation against various databases. Protein databases including Swiss-Prot UniProt (UniProt), Ensembl Plants (Plants) and NCBI NR (NR) were used for BLASTp annotation, and Pfam (Pfam) was used for Pfam Scan annotation. The number and percentage of common annotated genes are shown in the overlapping segment of the Venn diagrams. **c** Numbers of different transcript loci detected in the FL transcriptomes using the combination strategy. Cupcake, ‘*G. raimondii* genome as reference’; Cogent, ‘reconstructed gene sequences as reference’.

To reconstruct FL transcript models for *G. australe*, we developed a computational pipeline using several publicly available tools (Fig. 1a). High homology is known to exist between genes from *G. australe* and those from other *Gossypium* species, and COding GENome reconstruction Tool (Cogent) has limitations when analysing highly similar genes from large families [18]. Therefore, the isoforms were first collapsed using Cupcake based on a *G. raimondii* reference [19], and then Cogent was employed to reconstruct the resulting isoforms into one or several FL unique transcript model(s) based on the k-mer clustering and De Bruijn graph methods. As a result, we mapped 307,829 (96.09%) isoforms to the *G. raimondii* reference and collapsed 276,459 (86.30%) isoforms into 26,627 transcript loci by Cupcake. The remaining uncollapsed isoforms were presumed from *G. australe* species-specific sequences. Cogent was then subsequently employed to reconstruct the 43,898 remaining isoforms and generate 5165 ‘reconstructed gene sequences’. By applying the reconstructed gene sequences as a reference, we obtained 5274 transcript loci from the remaining isoforms. Finally, we collapsed 320,357 FL isoforms into 92,728 non-redundant isoforms derived from 31,904 loci.

Rarefaction analysis using subset of the FL reads revealed that sequencing depth was almost saturated for transcript loci discovery, implying that more sequencing data would not facilitate to discover much more transcript loci (Additional file 3:Figure S3a). While investigating on the relationship between sequencing depth and transcript discovery, we found that saturation reached slowly at the isoform level (Additional file 3:Figure S3b). This result reflected the overabundant transcripts in *G. australe* transcriptome, raising the possibility that lowly abundant or rare transcript isoforms remain to be discovered.

To verify the PacBio isoforms and remove isoforms with false splice junctions, we analysed splice motif from 79,390 (85.62%) non-redundant multi-exon isoforms. The results showed that a total of 177,349 splice junctions were captured from these multi-exon isoforms. Among them, most of the splice junctions (84.65%) were the canonical GT-AG, followed by non-canonical sites (GC-AG with 1.52%, AT-AC with 0.40%, and others with 13.43%) (Additional file 16:Table S3). To further validate the reliability of the above splice junctions detected in our Iso-Seq, RNA-Seq reads from leaf and seed germinated for 5 h, 15 h or 30 h were employed to map using STAR. The result demonstrated that 61.10–73.37% of the GT-AG were validated, followed by GC-AG (25.69–33.17%) and AT-AC (3.57–4.56%). None of the other non-canonical sites were validated by RNA-Seq. Totally, 82.08, 44.54 and 5.55% of GT-AG, GC-AG and AT-AC splice junctions in Iso-Seq were verified using RNA-Seq, respectively (Additional file 16:Table S3). The remaining non-supported splice junctions were presumed to be false splice junctions or tissue-specific expression of genes/transcripts. For example, the gene *PB.3866*, a seed-specific expression exon, was detected in RNA-Seq from seed germinated for 15 h and 30 h (Additional file 4:Figure S4). The isoform of *PB.3866* contains a GT-AG junction that was not supported by the *G. australe* leaf or seed RNA-Seq, inferring that this isoform might be tissue-specific expressed (Additional file 4:Figure S4). Thus, the splice junctions without RNA-seq-supported non-canonical motif were presumed as false splice junctions, and then the isoforms containing these false splice junctions were removed at last. Finally, 70,803 isoforms derived from 29,597 loci were for further analysed.

### Functional annotation of FL gene transcript loci in *G. australe*

Candidate open reading frames (ORFs) within the transcript sequences were detected by TransDecoder [20] with a minimum length of 100 amino acids (aa), and a log-likelihood score was calculated for each of the three reading frames. To maximize sensitivity for capturing ORFs with functional significance, we scanned all ORFs for homology to the Pfam protein domain database or the protein sequences curated in the NCBI NR (non-redundant proteins), Ensembl Plants and Swiss-Prot UniProt databases. When multiple ORFs appeared in a single transcript, the first ORF in each transcript was considered the ‘representative’. The analyses indicated the presence of 61,805 non-redundant isoforms (87.29% of the total) that contained high-quality ORFs with lengths ranging from 300 bp to 9345 bp with an average of 1171 bp, and these ORFs pertained to 25,246 transcript loci (85.30% of the total). Only 4, 351 transcript loci had no ORFs, inferred as non-coding RNAs, and these loci are addressed in the next section. The isoforms that contained the longest ORF in each transcript locus were employed as the ‘locus representative’ for subsequent comprehensive functional annotation.

To estimate the completeness of our transcriptome sequencing, we used BUSCO [21] for gene diversity estimation. BUSCO checks for essential single copy orthologs should be present in a whole transcriptome dataset for any member of the given lineage. The Embryophyta lineages (1440 conserved proteins) were used to examine transcriptome completion, in which the FL consensus isoforms and gene loci were used to ensure that completeness tracked across the collapsed steps. In our results for BUSCO alignments, the gene loci showed a lower completeness level than the FL consensus isoforms, being 80.42 and 91.04%, respectively (or 85.28 and 94.44%, respectively, when fragmented BUSCOs were counted) (Additional file 5:Figure S5). The FL consensus isoforms had a higher level of duplication in the BUSCO alignment, suggesting that the collapsed strategy successfully reconstructed transcript models and reduced duplicate isoforms from FL dataset. Finally, the entire representative CDS from each gene locus was aligned to the *G. raimondii* genome, and 22,733 (90.05%) loci were uniquely mapped onto 13 homologous chromosomes (Additional file 6:Figure S6). The number of genes located on each chromosome ranged from 1086 to 2851 (Additional file 6:Figure S6), and the numbers were positively correlated with chromosome length. In pericentromeric regions, which are known to be gene-poor and transposon-rich, very few genes were mapped. Most genes were distributed on the proximal ends in gene-rich regions (Additional file 6:Figure S6).

Most of the transcript loci (24,742, 98.00%) exhibited homology with at least one protein database, and 19,661 (77.88%) were found in all three protein databases (Fig. 1b). In addition, 20,543 transcript loci matched Pfam protein domains, and all but 16 of these loci exhibited homology with at least one protein database as well (Fig. 1b). Among the transcript loci collapsed in our analysis, 504 did not return any matches that contained an ORF; hence, they were considered potentially novel genes in *G. australe* (Additional file 17: Table S4). PlantTFDB [22] was employed to predict transcription factors from those gene loci. The results showed 1575 putative transcription factors from 55 families (Additional file 18:Table S5), representing 6.24% of the protein-coding genes. Then, using Gene Ontology (GO) and Kyoto Encyclopedia of Genes and Genomes (KEGG) software, we further assigned the genes to different functions. Finally, 18,722 (74.16%) and 5691 (22.54%) of the reconstructed genes were assigned to different functional terms in three GO categories and KEGG pathways, respectively (Additional file 17:Table S4). Among these GO categories, the functional terms ‘cellular process’, ‘metabolic process’ and ‘response to stimulus’ were identified to be the top three most common annotations among the ‘biological process’ GO terms (Additional file 7:Figure S7). Importantly, the *G. australe* genes annotated in ‘response to stimulus’ are valuable natural resources, which could be used to improve the resistance of cultivated species in future breeding programmes. In the KEGG annotated gene sets, the majority were classified into ‘Metabolism’, of which ‘Carbohydrate metabolism’, ‘Amino acid metabolism’ and ‘Lipid metabolism’ were the three most abundant pathways (Additional file 8:Figure S8). In particular, 270 (1.07%) *G. australe* genes belonged to ‘Metabolism of terpenoids and polyketides’. 13,005 single copy orthologs were detected by comparing with the *G. raimondii* [19], *G. arboreum* [23], *G. hirsutum* [24] (At and Dt subgenome) using OrthoMCL [25]. In addition, 7082 genes were found to be unique in *G. australe*. Functional enrichment analysis showed that these genes were highly enriched in ‘Biosynthesis of secondary metabolites’ in KEGG and ‘response to stimulus’ in GO. These genes would be employed to address some species-specific biological issues of *G. australe* in further research.

### Non-coding RNA analysis

To identify candidate non-coding RNAs from the *G. australe* PacBio data, 4351 (14.70%) non-ORF transcript loci were searched against the miRNA stem-loop sequences (28,645 records) curated in miRBase by BLASTN (E value <= 1e-5). In total, 86 transcript loci matched 65 pre-miRNA sequences (Fig. 1c). Among them, 28, 12 and 2 matched miRNAs from *G. raimondii*, *G. hirsutum* and *G. herbaceum*, respectively. The remaining 44 matched miRNAs from other species, suggesting that these are novel *Gossypium* spp. miRNAs (Additional file 19:Table S6). The remaining 4265 transcript loci out of 4351, which had lengths greater than 200 nucleotides, were subjected to lncRNA detection. Specifically, we used the longest isoform in each transcript locus as the ‘locus representative’ to validate its coding potential with CPC (Coding Potential Calculator) [26] and CNCI (Coding-Non-Coding Index) [27]. Finally, 1792 and 2415 of the loci were predicted as non-coding RNAs by CPC and CNCI, respectively (Additional file 9:Figure S9). Among them, 1468 common loci were predicted to yield non-coding RNAs by both programs, and these loci were considered lncRNAs (Fig. 1c and Additional file 20:Table S7). Only six of these 1468 lncRNAs were annotated with known non-coding RNAs (ncRNAs) by searching against ncRNAs in the NONCODE [28] database (Additional file 20:Table S7). To determine the functions of these lncRNAs, including the unannotated ones, further characterization must be performed experimentally.

### AS detection in the *G. australe* transcriptome

**Fig. 2:**
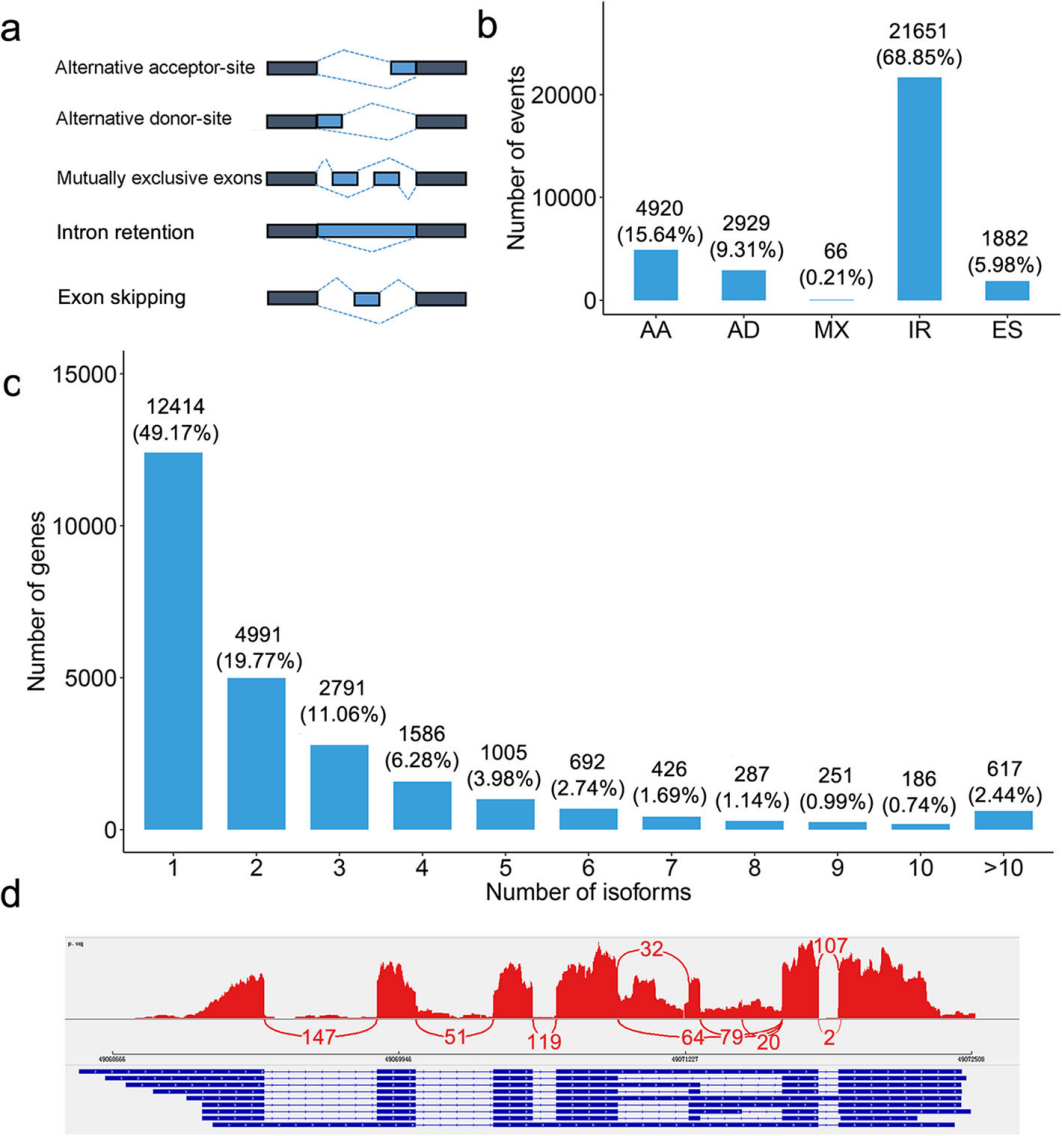
AS analysis of *G. australe* with Iso-Seq reads. **a** Visualization of five AS types. **b** The total number of AS events in *G. australe* based on Iso-Seq data. The number and percentage of AS events are shown at the top of each bar. **c** Distribution of genes that produce one or more splice isoforms in the Iso-Seq data. The number and percentage of genes are shown in the top of each bar. **d** Sashimi plot showing an example of a gene generating various transcript isoforms detected with our pipeline in the Iso-Seq data. There are nine different isoforms, including nine splice junction sites in Iso-Seq. Peaks in red represent short-read coverage, and curved lines with numbers in red represent splice junctions supported by that number of short reads. For each isoform, the blocks in blue represent exons, and the lines between the blocks represent introns

AS of precursor mRNAs (pre-mRNAs) from multi-exon genes allows organisms to increase their coding potential and regulate gene expression through multiple mechanisms [29–33]. Approximately 60% of protein-coding genes in plants contain introns that are excised to generate mature transcripts [29–31]. Previous reports have shown that plants have much more complex transcriptomes and that their FL transcripts are more sensitive than RNA-Seq assemblies for AS discovery, especially for genes with isoform complexity [8, 12]. PacBio Iso-Seq has proven to be a powerful tool for detecting complex AS events at the whole-transcriptome scale [34, 35]. Based on the reconstructed transcript loci, we found that 12,414 (49.17%) loci generated a single isoform, and approximately 12,832 (50.83%) genes produced two or more isoforms, while 803 (3.18%) genes had ten or more splice isoforms detected (Fig. 2c). Then, AStalavista [36] was used to ascertain the frequencies of the five main types of AS (exon skipping, ES; alternative acceptor site, AA; alternative donor site, AD; mutually exclusive exons, MX; and intron retention, IR) in the *G. australe* transcriptome (Fig. 2a). A total of 31,448 AS events were identified from 9944 genes in the Iso-Seq dataset (Additional file 21:Table S8), among which IR was the most abundant, accounting for 68.85% of AS events (Fig. 2b). Our result is consistent with those of previous reports showing that IR was the most frequent type of AS event in plants [30, 31, 37–42]. IR, AA (15.64%) and AD (9.31%) together accounted for more than 90% of the detected AS events in the *G. australe* transcriptome (Fig. 2b). For example, the gene *PB.15419* showed nine different isoforms by Iso-Seq, including nine splice junction sites. All of these isoforms were confirmed by RNA-Seq from leaf tissue, and eight of the junction sites were strongly supported by at least 20 RNA-Seq short reads (Fig. 2d). GO enrichment analysis showed that these AS genes were highly enriched in ‘phosphorus metabolic process’, ‘organelle organization’, ‘protein modification process’, ‘organonitrogen compound catabolic process’, ‘dephosphorylation’ and so on (Additional file 10:Figure S10). Moreover, genes with detected AS were highly enriched in the GO terms of several important metabolic processes associated with ‘spliceosome’ (Additional file 10: Figure S10), which plays a central role in catalysing pre-mRNA splicing.

### Alternative polyadenylation in *G. australe*

Alternative polyadenylation of the 3′ end of most transcripts is an important post-transcriptional modification, regulating gene expression through multiple mechanisms in most eukaryotic [43, 44]. Alternative polyadenylation caused by alternative cleavage increases transcriptome complexity. As full-length transcripts detected by Iso-Seq contain a poly(A) tail, it has advantages in the detection of alternative polyadenylation sites. Of the 25,246 genes detected by Iso-Seq, 14,536 genes have at least one poly(A) site, and 1075 genes have at least five poly(A) sites (Additional file 11:Figure S11a and Additional file 22: Table S9). The average number of poly(A) sites per gene was 2.16. Nucleotide composition flanking all poly(A) sites was analysed. There was clear nucleotide bias around poly(A) sites in *G. australe* with an enrichment of uracil (U) (upstream) and adenine (A) (downstream) (Additional file 11:Figure S11b). This nucleotide bias near poly(A) sites was consistent with previous reported [12, 13], suggesting that the identified poly(A) sites are credible. In addition, we performed MEME [45] to identify potential motifs enriched upstream of cleavage site. One conserved motif (AAUAAA) was identified around 25 nts upstream of the poly(A) site (Additional file 11:Figure S11c and d). This conserved motif is a known *cis*-elements as poly(A) signals in both plants and animals [43, 46].

### Differential expression analysis during germination

To better understand the transcript dynamic during seed germination, pairwise comparison was carried out and significance was confirmed using edgeR [47]. In total, 12,993 genes were assigned as differentially expressed genes (DEGs), with an FDR cutoff of 0.001 and |log2(fold change)| ≥ 1. The result indicated that DEGs between germinated seed (5 h vs 30 h) were more than that between germinated seed (5 h vs 15 h and 15 h vs30h) (Additional file 12:Figure S12a). Totally, 6956 DEGs were identified from pairwise comparison during germinating seeds; and 12,613 DEGs were detected between germinating seed and leaf (Additional file 12:FigureS12b). *G. australe* seed did not appear pigment glands at 5 h germination while glands appeared at 30 h. We then performed GO enrichment to analyze the function of 3643 DEGs overlapped between 5 h vs 30 h and 5 h vs 15 h (Additional file 12:Figure S12b). Our result showed that 10 GO-slim terms were identified as over-representations based on an FDR < 0.05, such as thylakoid, transmembrane transporter activity, carbohydrate metabolic process, extracellular region and etc. (Additional file 12:Figure S12c). To further illustrate the relationships among DEGs with highly similarities of temporal expression patterns, 12,993 DEGs were clustered into four main groups (designated K1–4) using K-mean clustering algorithm in TCseq package (Additional file 12:Figure S12d). The genes in each group enriched in particular functional categories. Four terms were significantly enriched in K1, they are ‘chromosome organization’, ‘organelle organization’, ‘cellular component organization’ and ‘cell wall organization or biogenesis’. Genes in K2 were identified responsible for cell cycle and cell division. K3 was suggested to be involving in ‘photosynthesis’, ‘lipid metabolic process’ and ‘response to stress’. K4 group contained the most overrepresented GO terms in four groups, in which genes involving in “macromolecule biosynthetic process”, “peptide metabolic process”, “cellular biosynthetic process” and “gene expression” were greatly enriched. However, the functional categories of secondary metabolic process and sesquiterpene biosynthetic process were not enrichment in these clusters.

### AS dynamics during seed germination in *G. australe*

**Fig. 3:**
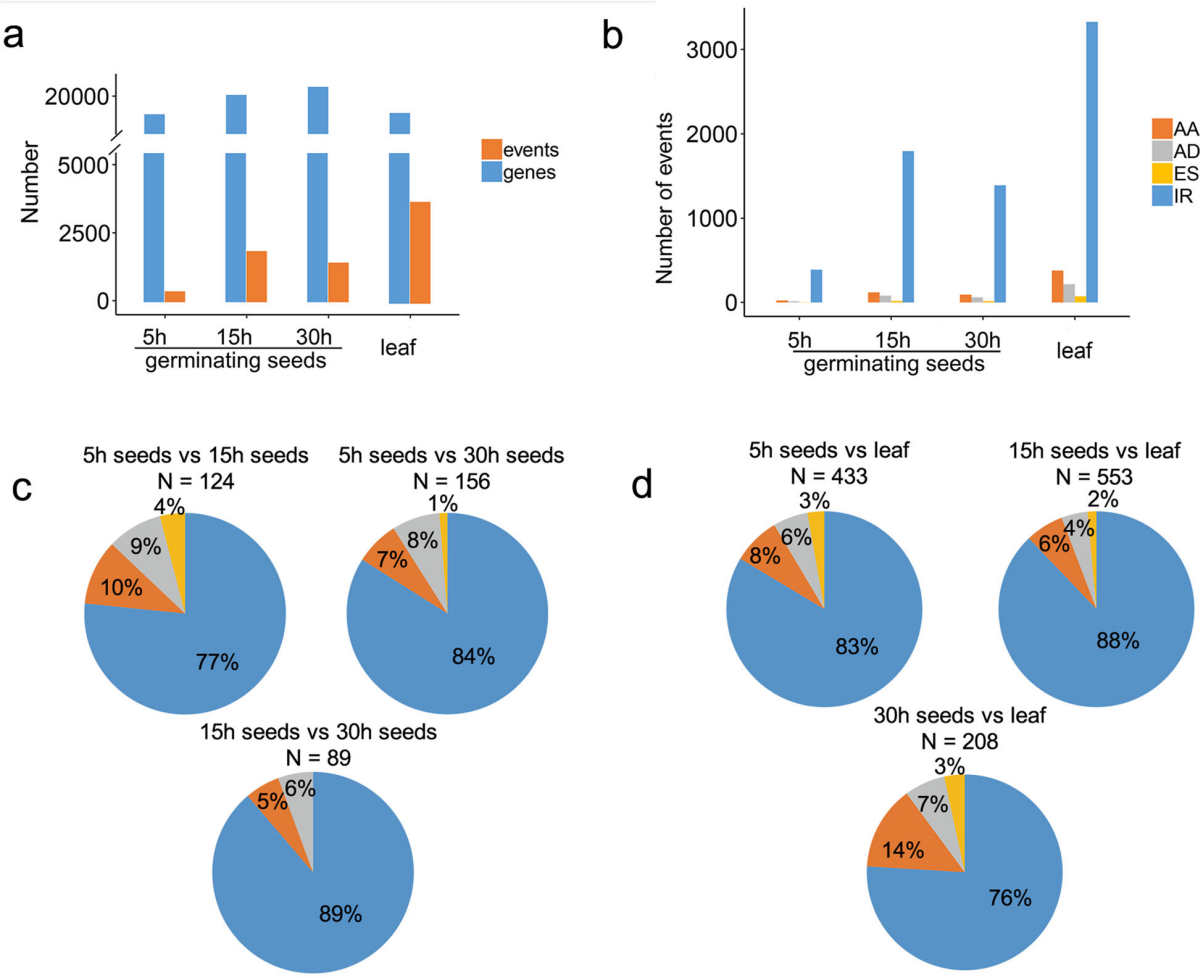
Distribution of AS events confirmed using RNA-Seq data in germinating seeds and leaf. **a** The number of expressed genes (FPKM > = 1.0) and events confirmed in germinating seeds and leaf. **b** The distribution of four AS types confirmed in germinating seeds and leaf. **c** The distribution of differentially expressed events detected through pairwise comparison in germinating seeds. **d** The distribution of differentially expressed events detected between germinating seeds and leaf. The pie chart represents the frequency of each AS event. N, number of differential events

Mature *G. australe* seeds are spotlessly white and without pigment glands. The pigment glands appear only after the seeds have germinated for more than 24 h [48]. To profile AS during the seed germination of *G. australe*, RNA-Seq paired-end reads during seed germination were employed to confirm the four major AS types (IR, AA, AD and ES) based on MISO [49]. The results demonstrated that the number of expressed genes (FPKM > = 1.0) rose slightly during seed germination, from 18,601 at 5 h to 20,616 at 30 h (Fig. 3a). The expressed gene number in the leaf (18,755) was slightly less than that in the germinating seed (Fig. 3a). Meanwhile, we found that the number of identified AS events increased during seed germination. In particular, many more AS events appeared in the leaf (3996), although the total number of gene loci was lower than that in the germinating seeds, indicating that AS plays an important regulatory role in the leaf tissues. In total, 4777 AS events were detected from 3338 genes in the four tissues. Among the four major AS types, IR and ES events were the most and the least frequent in all tissues, respectively (Fig. 3b). AA events were more frequent than AD events (Fig. 3b). These results are consistent with our Iso-Seq results (Fig. 2b). However, the percentage of IR detected by RNA-Seq was higher than that by Iso-Seq, accounting for over 80% of the total events (Fig. 3b). To characterize differentially expressed events during seed germination, we performed a pairwise comparison using MISO. In total, 124, 156 and 89 differential events were identified from 111, 139 and 86 genes, respectively, in the pairwise comparisons (at 5 h, 15 h and 30 h during germination) (Additional file 23:Table S10). The most abundant splicing difference was found between 5 h and 30 h of germination (Fig. 3c). Meanwhile, more differential AS events were present between 5 h and 15 h than between 15 h and 30 h, and this result is consistent with the finding that the number of detected AS events increased between 5 h and 15 h (Fig. 3a). No differential ES events were detected between 15 h and 30 h. We also performed a pairwise comparison based on tissues in the leaf and germinating seed (Fig. 3d), which showed 433, 553 and 208 differential events from 379, 496 and 184 genes, respectively (Additional file 23:Table S10).

**Fig. 4:**
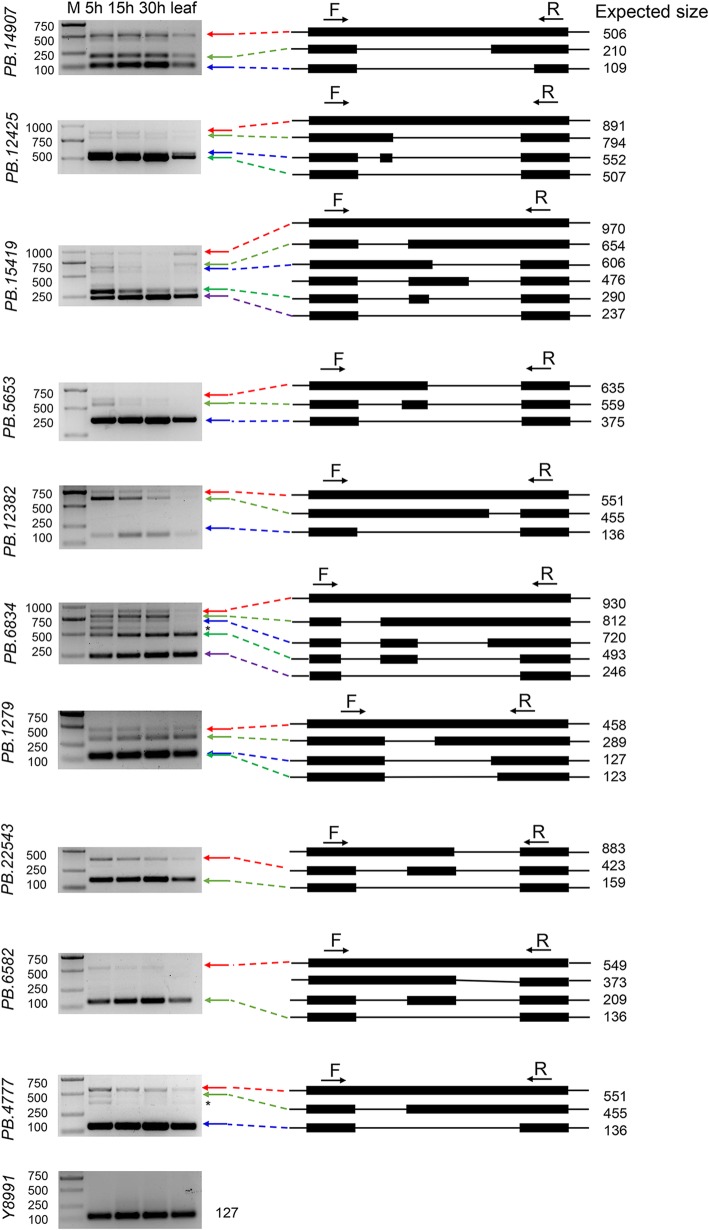
RT-PCR validation of AS events for ten genes. cDNA from 5 h, 15 h and 30 h germinating seeds and leaf samples were used for RT-PCR. Primer sets flanking the splicing events (F, forward and R, reverse) were designed. The gel bands in each figure show DNA markers and the RT-PCR results from four tissues/samples. The splice structure of each gene is shown in the right panel. Black boxes show exons, and lines show introns. The expected sizes are shown beside the structures. Asterisk indicates the isoform did not appear in Iso-Seq

To validate the robustness of the AS events detected, we randomly selected ten annotated genes to confirm their gene expression in germinating seeds and young leaves by semi-quantitative RT-PCR. Our RT-PCR indicated that the gel banding pattern and the size of the fragments were consistent with the AS events identified from the Iso-Seq data (Fig. 4). We found that the expression level of each isoform exhibited a tissue- and/or development-biased pattern. For instance, a small transcript fragment from the gene *PB.15419* (SNF1-related protein kinase regulatory subunit gamma-1) was preferentially expressed in 5 h germinating seeds (expected size, 290 bp). A fragment from the gene *PB.12382* (Pre-mRNA-processing factor) was expressed at the highest level in 5 h germinating seeds, gradually decreased during seed germination, and was not expressed in leaf (expected size, 455 bp). Overall, the results indicated that Iso-Seq was a powerful tool for the exploration of tissue-specific expression AS events/isoforms in combination with RNA-Seq data sets in the transcriptome.

### Re-characterization of genes involved in terpene biosynthesis in *G. australe*

**Fig. 5:**
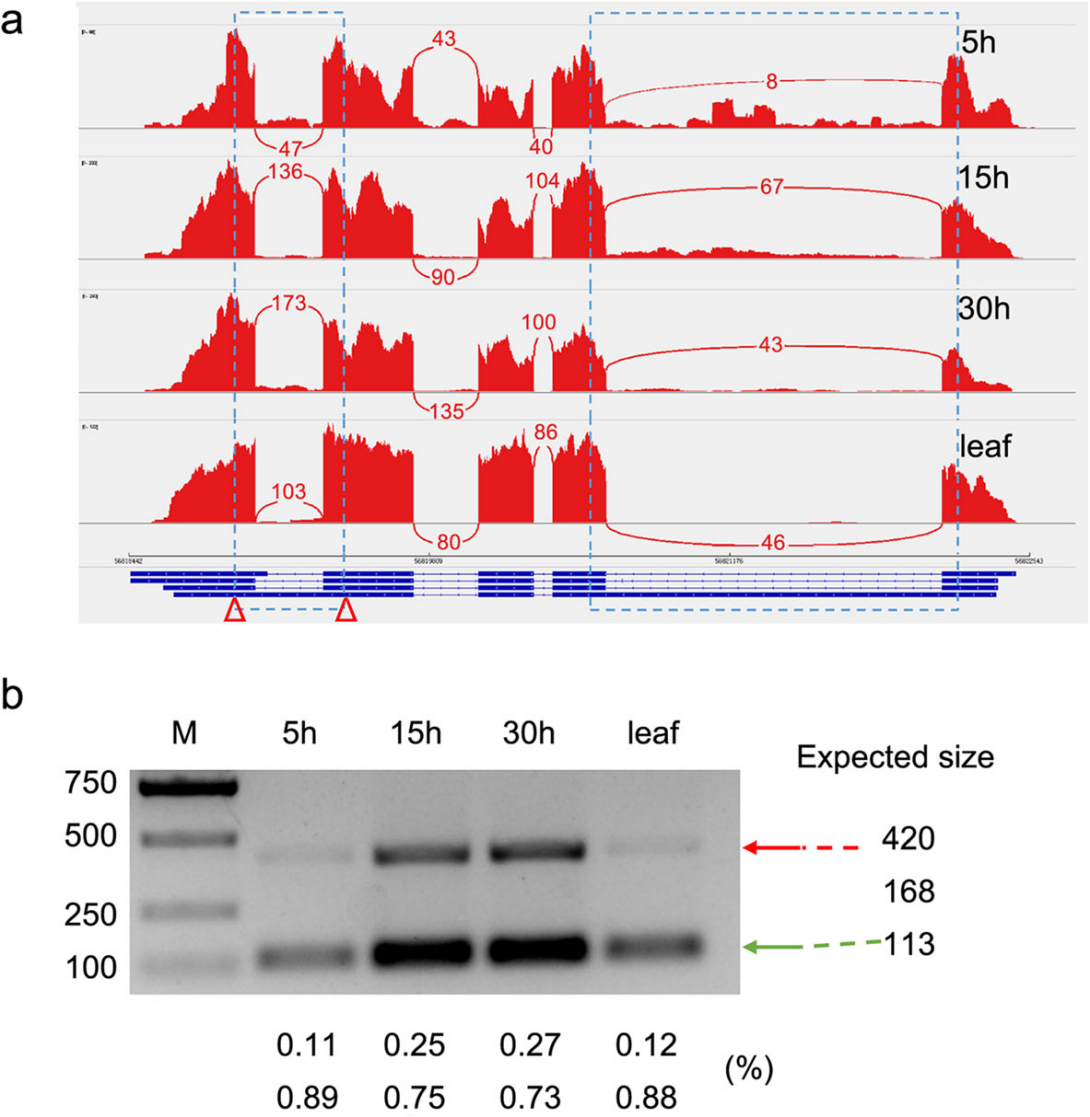
Sashimi plot and RT-PCR showing transcript isoforms of *PB.13680* (MK). **a** Sashimi plot shows the intron retention level of *PB.13680*. Peaks in red represent short-read coverage. For each isoform, blocks in blue represent exons, and lines between blocks represent introns. Red triangles indicate the position of primers used for RT-PCR. **b** RT-PCR validation of the differential intron retention level of *PB.13680*. cDNA from 5 h, 15 h and 30 h germinating seeds and leaf samples were used for RT-PCR. The gel bands show DNA markers and the RT-PCR products from four tissues/samples. The expected sizes are shown in the right panel. The percent of isoforms relative expression are shown in the below panel

The molecular mechanisms of delayed gland morphogenesis in Australian cotton species are still poorly understood. Australian wild cotton contains immature lysigenous glands but no terpenoid aldehydes, and its pigment glands appear only after seed germination; thus, there is little gossypol deposited in the dormant seeds of the species [4]. Gossypol, a natural terpenoid aldehyde that is toxic to humans and monogastric animals, reduces the value of cottonseed despite its richness in oil and proteins. The key genes involved in the gossypol biosynthetic pathways have already been identified [48, 50]. Accordingly, we manually investigated isogenes encoding the enzymes involved in both the methylerythritol phosphate (MEP)- and mevalonate (MVA)- dependent biosynthetic pathways, as well as a potential cotton delta-cadinene synthase (EC 4.2.3.13) in the *G. australe* FL transcriptome. We found that 38 genes were involved in gossypol synthesis in the MEP- and MVA-dependent biosynthetic pathways. Then, the expression of these genes were investigated in germinating seeds or leaf by RNA-Seq. Our results revealed that genes coding the main rate-determining enzyme of the MEP-pathway, 1-deoxy-D-xylulose 5-phosphate synthase (DXS), were detected with low expression level in germinating seed. Otherwise, genes coding the enzymes involving in MVA pathway were abundance (Additional file 13:Figure S13). The high expression levels of MVA pathway genes in 5 h and 15 h germinating seed indicated that gossypol biosynthesis occurred early even the seeds surface without pigment glands detected by naked eyes. In addition, most gene loci (30, 78.95%) had multiple isoforms in *G. australe* (Additional file 24:Table S11). Moreover, the intron retention level of *PB.13680*, which encodes MK (mevalonate kinase), gradually decreased during seed germination in *G. australe* (Fig. 5a). The IR event from *PB.13680* (the left border) was differentially expressed between the 5 h germinating seed and leaf. To validate the accuracy of the differential expression results, we designed primers for semi-quantitative RT-PCR at the region shown by a triangle in Fig. 5a. This gene could transcribe three isoforms, two of which were detected. We found that the fragment produced by an IR event was preferentially expressed in 15 h and 30 h germinating seeds (Fig. 5b). This result may suggest that gossypol biosynthesis is not only associated with gene expression but may also be regulated by different transcript isoforms resulting from AS.

## Discussion

### Challenges in the reconstruction of the *G. australe* FL transcriptome without a genome reference

Large-scale sequencing of cDNA is instrumental for transcriptome reconstruction and annotation, particularly for species without a reference genome sequence [9], but limitations in sequencing technologies frequently force trade-offs between sampling depth and completeness. Obtaining expressed sequence tags (ESTs) from cDNA libraries using Sanger sequencing was the first established transcriptome profiling method and has historically represented the gold standard for genome annotation projects. However, the high cost and time required for plasmid cloning and PCR amplification make this method impractical for larger sequencing projects. More recently, low-cost RNA-Seq has become available based on the development of next-generation sequencing techniques and has provided deep insights into gene expression analysis, alternative gene splicing characterization and single-nucleotide polymorphism identification [51]. However, RNA-Seq short-read libraries cannot provide complete transcripts, and transcript mis-assembly from RNA-Seq short reads by biosoftware and algorithms introduces inaccuracy to gene structure characterization [52], further impairing the accurate prediction of gene models for species without a reference genome sequence. Therefore, for transcriptome analysis in species without a reference genome sequence, long-read technologies are required to address the multiple requirements of scale, sampling depth, transcript completeness and cost.

In previous studies, a standard Iso-Seq bioinformatics pipeline has been developed and applied to transcriptome reconstruction in different species depending on the availability of genome assemblies [8, 12, 38]. However, there is still a lack of unified and effective approaches to Iso-Seq reconstruction without a reference. Recently, a tool, Cogent, for reconstructing the coding genome using high-quality Iso-Seq data was released [18]. Cogent is designed to be used in cases where no reference genome is available or the reference genome is highly incomplete. High homology is known to exist between genes from closely related species, and with the progress of genomic technology, a large number of species have been genome-sequenced. Here, we used a combination of ‘has genome’ and ‘no genome’ strategies to reconstruct our Iso-Seq data, and we obtained a comprehensive transcriptome atlas of *G. australe*. First, 25,246 gene loci were generated from our Iso-Seq data, providing a valuable ‘plug-and-play’ resource that can be directly used for future research in gene discovery, molecular breeding and delayed gland morphogenesis in *G. australe*. However, based on previous reports, there are ~ 40,000 protein-coding genes in diploid cotton such as A (*G. arboreum*) [23] or D (*G. raimondii*) [19] genome species. Our PacBio Iso-seq discovered about 63% of diploid cotton gene loci, which is similar to the previous reports in *G. barbadense* [38], *Zea mays* [8] and sugarcane [34]. The remaining undetected gene loci, we supposed, may be tissue specific- and/or stress-induced transcribed. In addition, our previous study found that *G. australe* was jresistant to Verticillium wilt [53]. Therefore, to capture pathogen-induced genes, the roots, stems and leaves from seedlings inoculated with *V. dahliae* were also used for further discovery of genes resistant to diseases. Second, taking a comprehensive view, we first identified splice variants at the whole-genome level in *G. australe* based on the effective coding genome. More than half of the genes had two or more isoforms, suggesting a high degree of transcriptome complexity. Combining these alternative splicing annotation with RNA-Seq results from individual tissues, we found differential expression of AS events, which probably corresponded to tissue- and development-specific FL splice isoforms. Sequencing of the *G. australe* transcriptome with RNA from different tissues and under diverse stress conditions will allow us to discover tissue- or development-specific and stress-regulated splice variants. Finally, we detected 86 pre-miRNAs and 1468 lncRNAs in our Iso-Seq data. These results provide a useful resource for understanding the potential functions of miRNAs and lncRNAs in *G. australe*. Overall, obtaining the desired results in *G. australe* not only demonstrates that this strategy can be used to reconstruct coding genomes via Iso-Seq in species lacking a reference genome but also provides a novel, valuable genetic resource for future research on reducing cottonseed gossypol content and illuminating the relationships between gossypol synthesis and gland morphogenesis.

### Relationships between gossypol synthesis and gland morphogenesis

Approximately 10–11 million metric tons of cottonseed protein are annually produced worldwide, which would be enough to satisfy the daily basic protein needs (50 g/person) of more than 600 million people each year [54]. Elimination of gossypol from cottonseed could greatly improve the utilization of this valuable protein resource and, thus, assist in ensuring global food and fibre security as we move into an era of uncertain climate conditions. To achieve these goals, scientists have undertaken numerous studies in the fields of traditional and molecular breeding [4, 54, 55].

Pigmented lysigenous glands located throughout the cotton plant represent significant repositories of gossypol. At least six independent loci, *gl*_1_, *gl*_2_, *gl*_3_, *gl*_4_, *gl*_5_ and *gl*_6_, are known to jointly determine cotton gland formation [3]. Moreover, two pairs of duplicate recessive genes (*gl*_2_ and *gl*_3_), located on chromosomes (chrs.) A12 and D12, respectively, mainly maintain the glandless phenotype. Additionally, a single dominant glandless mutant (*Gl*_2_^e^) was obtained by irradiation of seeds from *G. barbadense* var. Giza 45 with ^32^P in Egypt. Our previous report identified a bHLH transcription factor (*GoPGF*) as the causative gene for the glandless phenotype in cotton, and this gene also appeared to be a regulator of glandular trichome formation [3]. When *GoPGF* was silenced by VIGS, the gossypol content and pigment glands in the cotton plant greatly decreased simultaneously. However, the molecular mechanism of the delayed gland morphogenesis trait in Australian *Gossypium* species such as *G. australe*, *G. bickii* and *G. sturtianum* remains obscure*.* Intriguingly, *G. stocksii*, an E genome species, exhibits a unique characteristic; its dormant seeds are covered with glands but lack gossypol production. Therefore, it is reasonable to infer that gossypol synthesis and gland morphogenesis were controlled by different pathways. Based on the relation between gossypol synthesis and gland morphogenesis, we conjecture that gossypol synthesis is dependent on gland morphogenesis, while gland morphogenesis is independent of gossypol synthesis. That is, if no pigment glands are present on a cotton plant, it will scarcely synthesize gossypol, resulting in very low gossypol content (as seen in the pigment-gland mutants of upland cotton and Australian C and G genome cotton species). In addition, if pigment glands do develop on a cotton plant, it will normally synthesize gossypol (e.g., cultivated cotton and most of the wild species) but may not (e.g., several wild species such as *G. stocksii*). Even so, the precise mechanisms of gossypol synthesis and gland morphogenesis remain to be elucidated.

*G. australe* possesses the delayed gland morphogenesis trait, having glanded plants but glandless seeds. Thus, this species differs from upland cotton, which is either glanded or glandless in both plant and seed. Here, we manually investigated isogenes encoding the enzymes involved in both the MEP- and MVA-dependent biosynthetic pathways, as well as a potential cotton delta-cadinene synthase (EC 4.2.3.13). Finally, we found 38 genes involved in gossypol synthesis in the MEP- and MVA-dependent biosynthetic pathways, and most of these gene loci (30, 78.95%) had multiple isoforms in *G. australe* (Additional file 24:Table S11). These results should facilitate to understand the relation between gossypol synthesis and gland morphogenesis. In the future, our task will be focused on identification of additional candidate genes via transcriptome comparison between *G. australe* and other glanded-seed species such as *G. arboreum* and via coexpression analysis with the bait *GoPGF*. Previous ultrastructural studies on glands have shown that the formation of pigment glands is a lysigenous process and that programmed cell death (PCD) plays a critical role in the development of pigment glands in *Gossypium* [56]. In contrast to the glandless mutation in *G. hirsutum*, the glandless seeds of wild Australian species have special cells called the “gland primordium”, although these cells are invisible to the naked eye [57]. Thus, we hypothesize that seed pigment gland development in *G. australe* ceases at the gland primordium stage because abnormal PCD makes it impossible for lysigenous development to proceed successfully. Whether PCD plays a critical role in delayed gland morphogenesis, however, remains to be elucidated in future research.

## Conclusions

In this study, PacBio Iso-Seq was employed to generate *G. australe* transcript data from ten tissues. Finally, a total of 1,001,928 CCS collapsed into 92,728 non-redundant isoforms, these isoforms derived from 31,904 transcript loci. Rarefaction analysis using subset of the FL reads revealed that sequencing depth was almost saturated for transcript loci discovery. 25,246 transcript loci (85.30%) contained high-quality ORFs (≥100 aa), accounting for about 63% of the total predicted genes in the diploid cotton. We first identified splice variants in *G. australe* at the whole-genome level based on the effective coding genome. More than half of the genes had two or more isoforms, and 31,448 AS events were identified in the Iso-Seq dataset, among which IR was the most abundant, accounting for 68.85% of AS events. 29,718 polyadenylation sites were detected from 14,536 genes, 7900 of which have alternative polyadenylation sites. Combining these alternative splicing annotation with RNA-Seq results from germinating seeds, we found differential expression of AS events, which probably corresponded to tissue- and development-specific FL splice isoforms. This full-length transcript dataset provides a novel, valuable genetic resource for understanding the mechanisms underlying the complexity of alternative splicing and alternative polyadenylation in *G. australe*.

## Methods

### Plant materials

The *G. australe* tissues used in this experiment, 0 dpa (days post anthesis) flowers and 5, 10 and 15 dpa capsules, were collected from Pailou Experimental Station of Nanjing Agricultural University (PES/NAU) for PacBio sequencing. Under controlled chamber conditions (day/night, 28/26 °C, 16/8 h), 14-day-old seedlings were inoculated with ddH_2_O or the highly aggressive defoliating *V. dahlia* strain V991 (kindly provided by the Institute of Plant Protection of Jiangsu Academy of Agricultural Sciences) with 20 ml conidial suspension (1 × 10^7^ conidia/ml) per pot. Four days later, we collected samples (root, stem and leaf) and immediately placed them into liquid nitrogen. Finally, in the laboratory, the samples were removed from the liquid nitrogen and kept in a freezer at − 80 °C until use.

### PacBio Iso-Seq library preparation and sequencing

Using the TRIzol reagent (Life Technologies, Carlsbad, CA, USA, No. 15596–026), total RNA was isolated from each frozen tissue according to the manufacturer’s instructions and checked for integrity using a Bioanalyzer (Agilent, https://www.agilent.com/). The Iso-Seq library was prepared according to the Isoform Sequencing protocol using the Clontech SMARTer PCR cDNA Synthesis Kit and the BluePippin Size Selection System protocol as described by Pacific Biosciences (PN 100–378–900-01 and P/N100–377–100-05) with the following modifications. One microgram of equally mixed RNA from 10 different tissues was used for input into the Clontech SMARTer reaction. Amplification of cDNA with 12 cycles was performed using Phusion High-Fidelity DNA Polymerase (#M0530; NEB, http://www.neb.com). The amplification products were then size-selected using the BluePippin (Sage Science, http://www.sagescience.com/) with the following bins for each sample: <= 4 kb and > 4 kb. After size selection, large-scale PCR was performed using the eluted DNA from the previous step with 12 PCR cycles to generate more double-stranded cDNA for SMRTbell library construction. The amplified and size-selected cDNA products were made into SMRTbell Template libraries according to the Iso-Seq protocol referenced above. The libraries were prepared for sequencing by annealing a sequencing primer (component of the SMRTbell Template Prep Kit 1.0) and binding polymerase to the primer-annealed template. Five SMRT cells were sequenced on the PacBio Sequel platform using P6-C4 polymerase (Pacific Biosciences, P/N 100–372-700) and chemistry (Pacific Biosciences, P/N 100–356-200).

### Transcriptome analysis pipeline for isoform sequencing

First, we divided the Iso-Seq raw reads into CCS and non-CCS subreads with the SMRT Link 4.0 software. Next, we determined which transcripts were FL using the poly (A) tail and 5′/3′ primer information from the cDNA kit. To improve consensus accuracy, CCS was executed by SMRT Link, and high-quality, FL, and polished consensus transcripts were obtained using the isoform-level clustering algorithm ICE (Iterative Clustering for Error Correction). Finally, RNA-Seq short reads from the *G. australe* leaf and seed were employed to correct sequencing errors in the consensus transcripts using the program LoRDEC [17]. The corrected consensus transcripts were mapped to the *G. raimondii* genome by gmap [58] (−-cross-species --max_introlength-middle = 20,000 --no-chimeras -n 0 --split-large-introns). Based on the mapping result, redundant isoforms were further collapsed into transcript loci by Cupcake (−c 0.8 -i 0.7) (https://github.com/Magdoll/cDNA_Cupcake); 5′ differences were not considered when collapsing the isoforms. The resulting isoforms were used to reconstruct the coding genome with Cogent (https://github.com/Magdoll/Cogent, v2.1).

### Gene prediction and annotation

ORFs for each transcript were detected by TransDecoder [20] with a minimum length of 100 amino acids (aa). Where multiple ORFs were found in a single transcript, we used the first ORF in each transcript as the ‘representative’. In each transcript locus, we defined the isoform containing the longest ORF as the ‘locus representative’ for functional annotation. Protein sequences were downloaded from Ensembl Plants (http://plants.ensembl.org/info/website/ftp/index.html, release 37), Swiss-Prot (ftp://ftp.uniprot.org/pub/databases/uniprot/current_release/knowledgebase /complete/uniprot_sprot.fasta.gz) and NCBI NR (ftp://ftp.ncbi.nlm.nih.gov/blast /db/FASTA/). We then removed the records for proteins described as hypothetical or predicted in these databases. As a result, we obtained 1,987,885 protein sequences in the Ensembl Plants database, 556,006 protein sequences in the Swiss-Prot database and 84,421,649 protein sequences in the NR database. We searched these well-characterized protein databases for the ‘locus representative’ protein sequences using BLASTp (NCBI-BLAST v2.2.28+) with the following parameters: -outfmt 6 -max_target_seqs 1 -evalue 1e-5. Meanwhile, the Pfam database of 16,712 protein families was downloaded from (ftp://ftp.ebi.ac.uk/pub/databases/Pfam, release Pfam31.0). Pfam Scan was used to identify occurrences of the known protein domains documented in the Pfam database with the parameters ‘E 1e-5’. Transcription factors were predicted using PlantTFDB v4.0 [22]. The GO terms and pathways in KEGG for each gene were obtained from KOBAS using *Arabidopsis thaliana* protein sequences as reference [59].

To classify the AS events, the tool AStalavista was employed using the raw.gtf files from Iso-Seq FL consensus transcripts collapsed. Five major AS types, namely IR (AS code: 1^2-,0), ES (AS code: 1–2^,0), AA (AS code: 1-,2-), AD (AS code: 1^,2^), and MX (AS code: 1–2^,3–4^), were extracted from the output files and counted, respectively. APA sites were identified using FLnc reads by TAPIS described previously [12]. MEME was used for motif searches on the flanking sequence of polyadenylation sites [45].

### Non-coding RNA

If there was no ORF or the ORF length was less than 100 amino acids in a transcript locus, the transcript locus was considered a putative non-coding RNA. The putative non-coding RNAs were aligned (blastn, e value 1e-5) to all plant miRNA stem-loop sequences from miRBase, a repository of miRNAs from several species (http://www.mirbase.org/, release 21), to identify pre-miRNAs. The remaining putative non-coding RNAs that were not pre-miRNAs and had lengths of more than 200 bp nucleotides were subsequently evaluated for coding potential with CPC v0.9r2 [26] and CNCI v2 [27] with default parameters. When they passed evaluation by both methods, the loci were annotated as lncRNAs. Finally, these lncRNAs were used to search Arabidopsis lncRNA sequences in the NONCODE database [60] using blastn (−evalue 1e-5).

### Construction of Illumina RNA-Seq library for sequencing

*G. australe* total RNA was extracted from frozen tissues using the Plant RNA Extraction Kit (Zoonbio Biotechnology, RK16-50 T) as described in the user’s manual. The integrity, quality and concentration of the extracted RNAs were assessed with the Agilent 2100 Bioanalyzer (Agilent Technologies, Waldbronn, Germany). The cDNA libraries of high-quality RNA samples were prepared using the Illumina mRNA-Seq sample preparation kit (Illumina, San Diego, CA) and sequenced by the HiSeq 2000 platform with standard protocols. Raw reads were cleaned by removing both adapter sequences and low-quality (Phred score < 20) sequences using Trimmomatic (http://www.usadellab.org/cms/?page=trimmomatic, Version 0.33). In addition, the transcriptome data of *G. australe* 5 h to 30 h germinating seeds were downloaded from NCBI (https://www.ncbi.nlm.nih.gov/bioproject/PRJNA212007/).

### Read alignment and RNA-Seq data analysis

Each of the *G. australe* cleaned data sets was aligned to the in silico reference (*G. raimondii* plus ‘reconstructed gene sequences’) by STAR [61] with the default parameters, and the aligned results were used for detecting junctions. MISO [49] was employed to verify AS events in RNA-Seq using the raw.gtf file from the SMRT sequencing data and the aligned results with the paired-end model. The insert length distribution of each sample was computed by utilizing ‘exon_utils’ and ‘pe_utils’ in the MISO software package. The AS events passed a filter with the parameters ‘-num-inc 1 -num-exc 1 -num-sum-inc-exc 10’, and those with confidence intervals (CIs) not more than 0.2 were considered to be present in the sample. Pairwise comparison was executed by ‘compare_miso’. The results that passed a filter defined by filter_events with parameters ‘-num-inc 1 -num-exc 1 -num-sam-inc-exc 10 -delta-psi 0.20 -bayes-factor 10’ were defined as differential events. The expression of each gene was determined using FPKM (fragments per kilobase per million mapped reads) with Cufflinks (version 2.2.1) [62].

### PacBio isoforms validation by RT-PCR

cDNA synthesis was performed using HiScript II Q RT SuperMix for qPCR (+gDNA wiper) (R223–01, Vazyme), 1 μg of total RNA was treated with 4 × gDNA wiper Mix and then used to synthesize the first-strand cDNA using SuperMix II primed with oligo dT. Reverse transcription (RT)-PCR was performed to validate the PacBio isoforms as described previously [63]. Primers were designed to span the splicing events using Primer Premier Software (v.5.0). The RT-PCR amplification products were separated and observed on 1% agarose gel. The endogenous *G. australe* histone3 gene (Y8991) was used as an internal standard. The primers used in validation are listed in Additional file 25:Table S12.

## Additional files

Additional file 1: **Figure S1.** High-quality RNAs from ten different *G. australe* tissues on Bioanalyzer. DPA, day post anthesis; DPI, day post inoculation. (TIF 9650 kb)

Additional file 2: **Figure S2.** Length distribution of consensus reads. (TIF 7185 kb)

Additional file 3: **Figure S3.** Rarefaction analysis with number of full-length reads. a. Rarefaction analysis of covered transcript loci. b. Rarefaction analysis of covered isoforms. (TIF 5790 kb)

Additional file 4: **Figure S4.** Sashimi plot showed the exons and junctions of the gene *PB.3866* in leaf and germinating seed RNA-Seq data. (TIF 5103 kb)

Additional file 5: **Figure S5.** Transcriptome completeness analysis based upon BUSCO alignment. (TIF 9063 kb)

Additional file 6: **Figure S6.** Distribution of *G. australe* genes along *G. raimondii* chromosomes. (TIF 9997 kb)

Additional file 7: **Figure S7.** GO term classification of *G. australe* genes. BP, biological process; CC, cellular component; MF, molecular function. (TIF 7061 kb)

Additional file 8: **Figure S8.** KEGG pathway classification of *G. australe* genes. (TIF 8819 kb)

Additional file 9: **Figure S9.** Coding potential evaluated by CPC and CNCI. (TIF 2364 kb)

Additional file 10: **Figure S10.** GO enrichment analysis of AS genes. (TIF 7532 kb)

Additional file 11: **Figure S11.** APA analysis. a. Distribution of the number of poly(A) sites per gene. b. Nucleotide composition around poly(A) cleavage sites. c. MEME analysis of an over-represented motif at 25-nts upstream of the poly(A) site. d. Density distribution of detected motif flanking poly(A) site in our Iso-Seq data. (TIF 9733 kb)

Additional file 12: **Figure S12.** Differentially expressed genes. a. Numbers of up- and down-regulated DEGs. b. Numbers of DEGs overlapped by pairwise comparison. c. GO enriched terms of 5 h vs 15 h and 5 h vs 30 h overlapped genes. d. Expression patterns of genes in the 4 clusters. e. Functional enrichment among the 4 clusters. (TIF 10128 kb)

Additional file 13: **Figure S13.** Expression levels of genes involved in MEP- and MVA-pathway. (TIF 9397 kb)

Additional file 14: **Table S1.** Summary of PacBio single-molecule long-read sequencing. (XLSX 9 kb)

Additional file 15: **Table S2.** Statistics of LoRDEC error correct results. (XLSX 8 kb)

Additional file 16: **Table S3.** Splicing junction validated by RNA-Seq. (XLSX 9 kb)

Additional file 17: **Table S4.** Functional annotation of the genes obtained from Iso-Seq. (XLSX 2815 kb)

Additional file 18: **Table S5.** Transcription factors identified in *G. australe*. (XLSX 72 kb)

Additional file 19: **Table S6.** MiRNA identified in *G. australe*. (XLSX 14 kb)

Additional file 20: **Table S7.** LncRNA identified and known RNA motifs annotated in *G. australe*. (XLSX 84 kb)

Additional file 21: **Table S8.** The alternative splicing events detected by Iso-Seq. (XLSX 2019 kb)

Additional file 22: **Table S9.** Genes with poly(A) sites. (XLSX 695 kb)

Additional file 23: **Table S10.** The differential alternative splicing events in 5 h, 15 h and 30 h germinating seeds and leaf samples. (XLSX 210 kb)

Additional file 24: **Table S11.** Re-characterizing transcription isoforms of genes involved in gossypol biosynthesis pathway. (XLSX 16 kb)

Additional file 25: **Table S12.** The primers used for RT-PCR analysis. (XLSX 9 kb)

## Data Availability

Raw data of Iso-Seq are available under NCBI BioProject database under accession number PRJNA560198. The Illumina RNA-Seq data of *Gossypium australe* germinating seeds were downloaded from NCBI under accession number PRJNA212007. All data generated or analyzed during this study are included in this published article and its supplementary information files.

## Abbreviations

AA: Alternative acceptor site
AD: Alternative donor site
APA: Alternative polyadenylation
AS: Alternative splicing
BUSCO: Benchmarking universal single-copy orthologs
CCS: Circular consensus sequences
cDNA: complementary DNA
CI: Confidence interval
CNCI: Coding-Non-Coding Index
CPC: Coding Potential Calculator
ES: Exon skipping
EST: Expressed sequence tag
FL: Full-length
FLnc: Full-length non-chimeric
GO: Gene ontology
ICE: Iterative clustering for error correction
IR: Intron retention
Iso-Seq: Isoform sequencing
KEGG: Kyoto encyclopedia of genes and genomes
MEP: Methylerythritol phosphate
MVA: Mevalonate
MX: Mutually exclusive exons
nFL: non-full-length
ORF: Open reading frame
PacBio: Pacific Biosciences
PCD: Programmed cell death
RNA-Seq: high-throughput RNA sequencing
RT-PCR: Reverse transcription-polymerase chain reaction
SMRT: Single-molecule real time
TF: Transcription factor
