# Supplementary figures and images for: Reconstruction of the full-length transcriptome atlas using PacBio Iso-Seq provides insight into the alternative splicing in Gossypium australe
Source: BMC Plant Biol. 2019 Aug 19;19:365. doi: 10.1186/s12870-019-1968-7 (PMC6701088; doi:10.1186/s12870-019-1968-7)

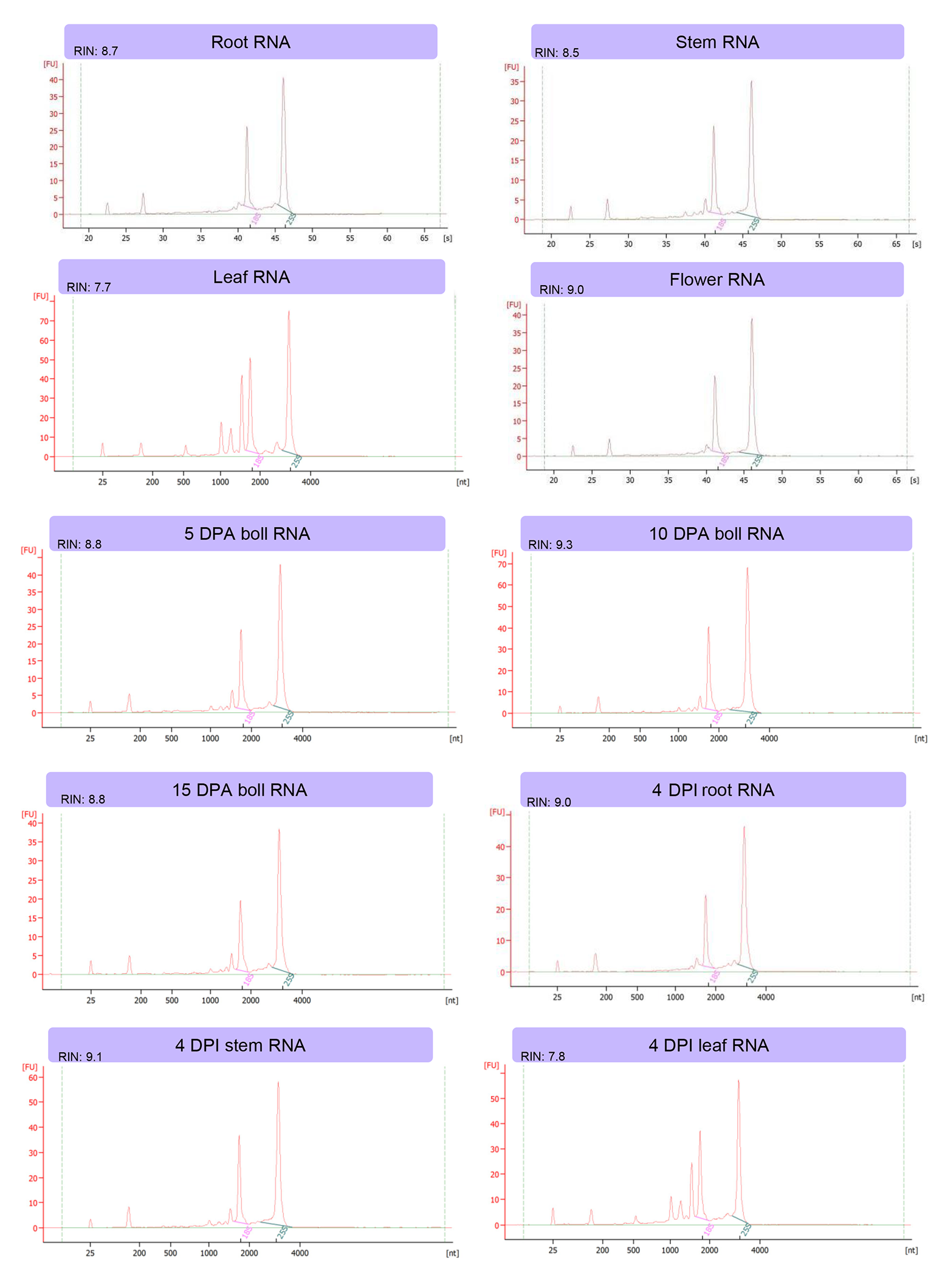

Supplement: Supplementary file 1 — Figure S1. High-quality RNAs from ten different G. australe tissues on Bioanalyzer. DPA, day post anthesis; DPI, day post inoculation. (TIF 9650 kb) [file 12870_2019_1968_MOESM1_ESM.tif]

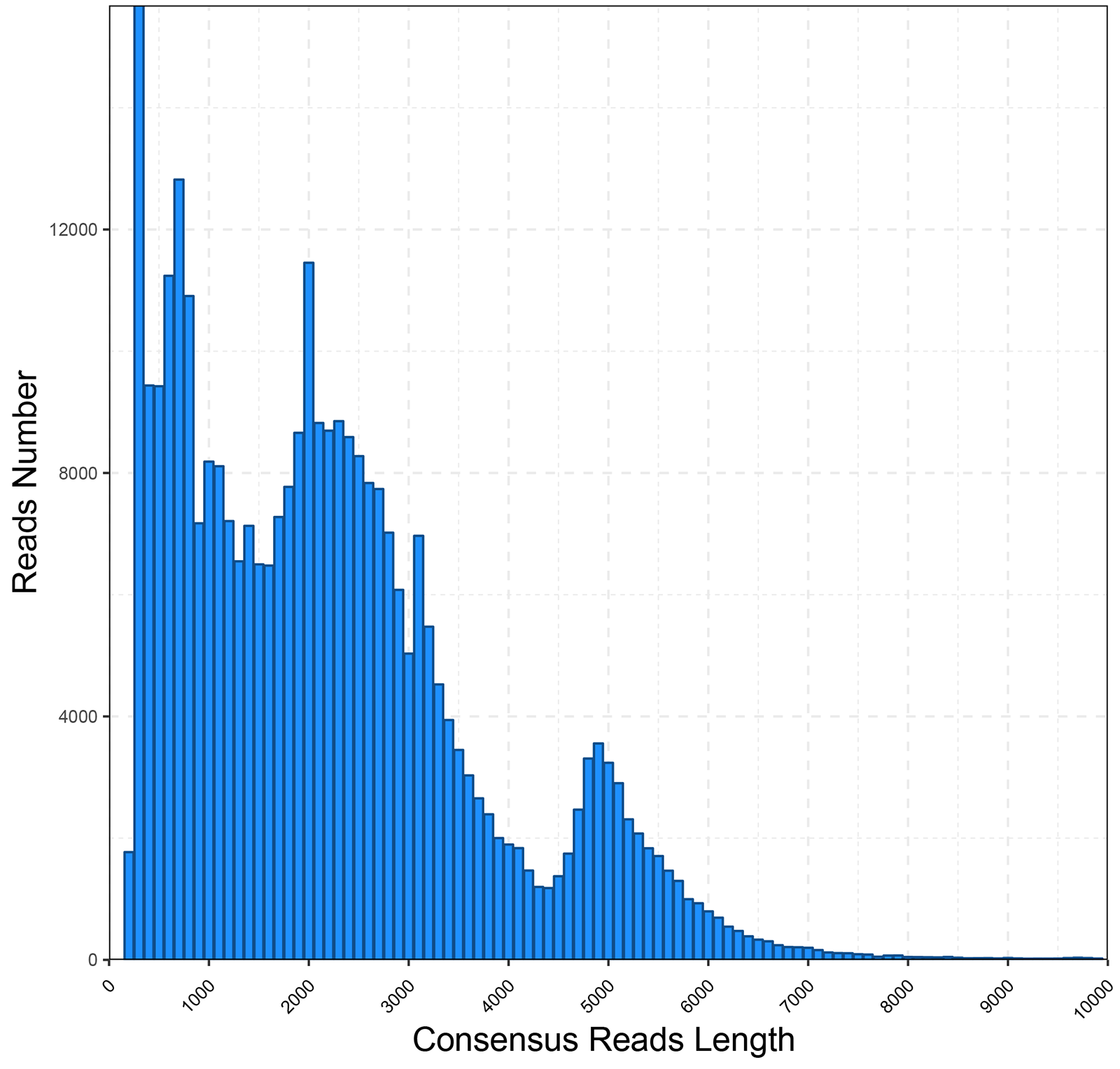

Supplement: Supplementary file 2 — Figure S2. Length distribution of consensus reads. (TIF 7185 kb) [file 12870_2019_1968_MOESM2_ESM.tif]

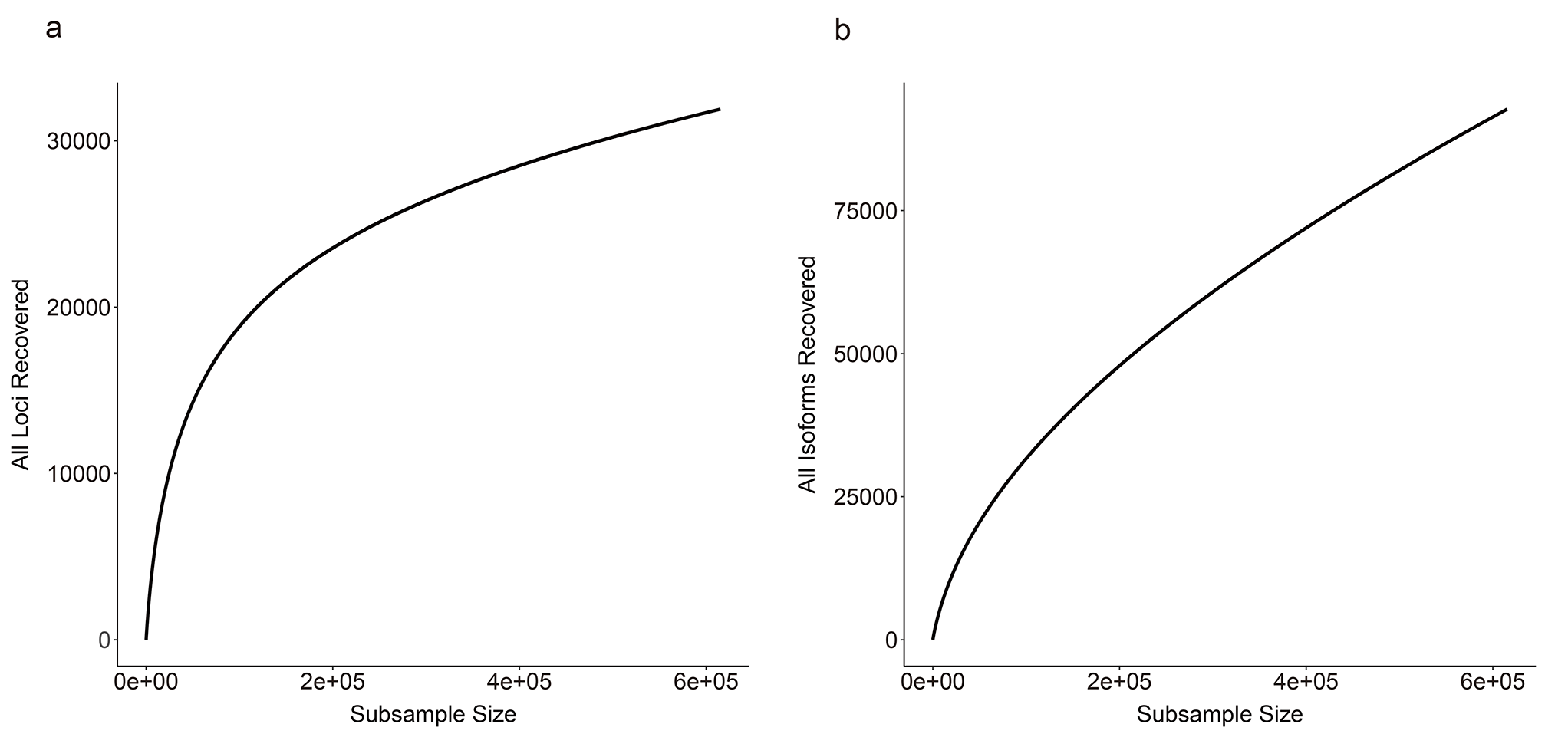

Supplement: Supplementary file 3 — Figure S3. Rarefaction analysis with number of full-length reads. a. Rarefaction analysis of covered transcript loci. b. Rarefaction analysis of covered isoforms. (TIF 5790 kb) [file 12870_2019_1968_MOESM3_ESM.tif]

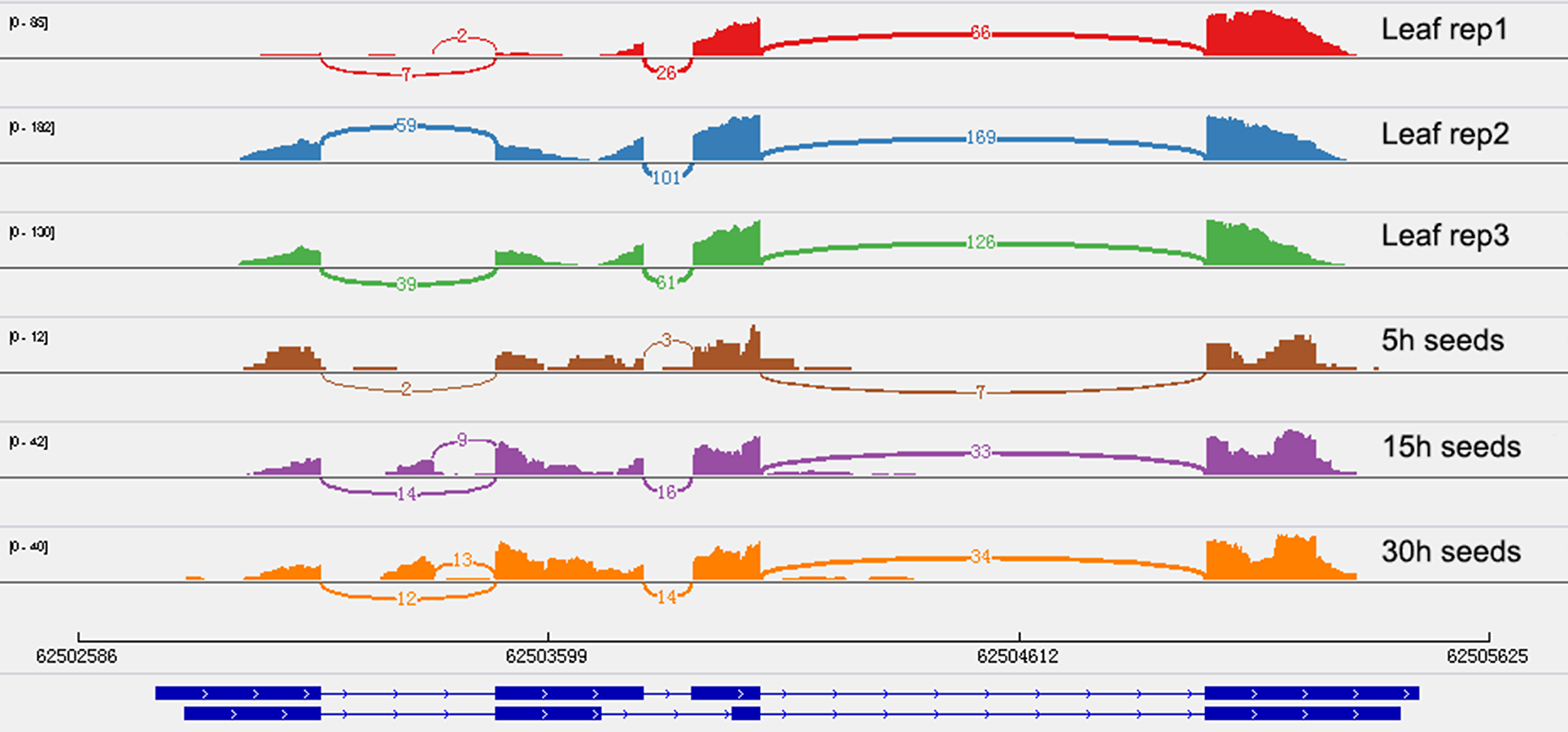

Supplement: Supplementary file 4 — Figure S4. Sashimi plot showed the exons and junctions of the gene PB.3866 in leaf and germinating seed RNA-Seq data. (TIF 5103 kb) [file 12870_2019_1968_MOESM4_ESM.tif]

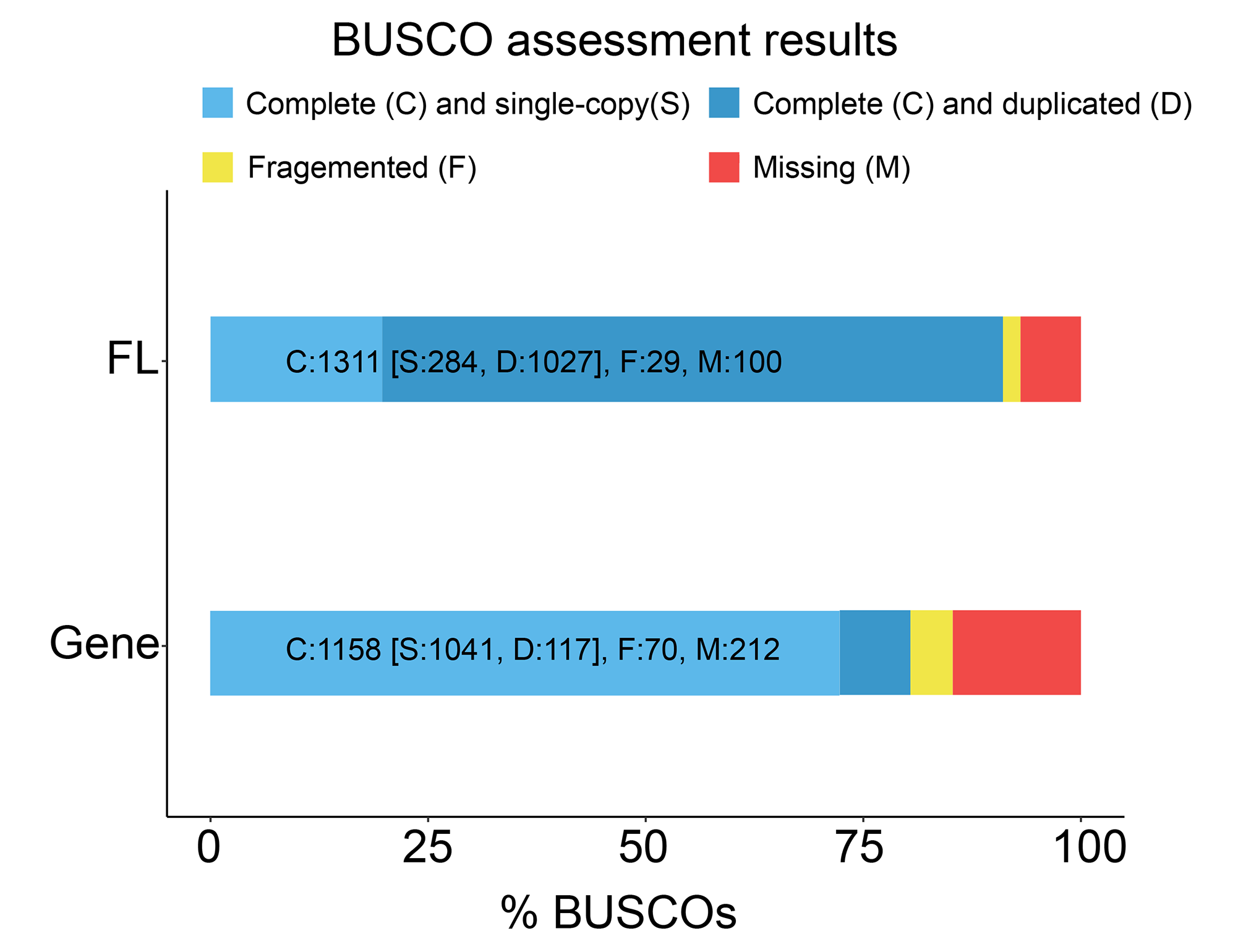

Supplement: Supplementary file 5 — Figure S5. Transcriptome completeness analysis based upon BUSCO alignment. (TIF 9063 kb) [file 12870_2019_1968_MOESM5_ESM.tif]

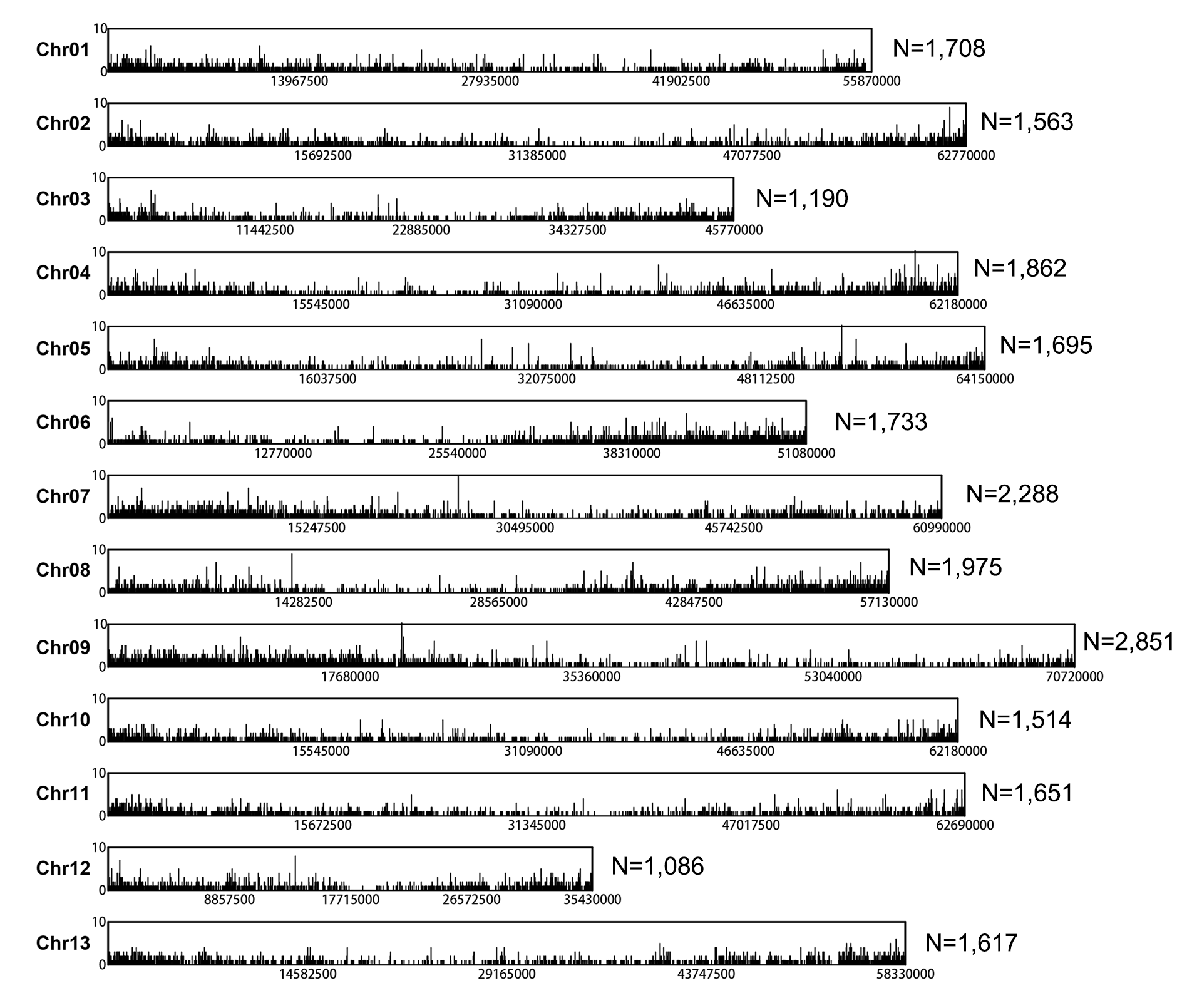

Supplement: Supplementary file 6 — Figure S6. Distribution of G. australe genes along G. raimondii chromosomes. (TIF 9997 kb) [file 12870_2019_1968_MOESM6_ESM.tif]

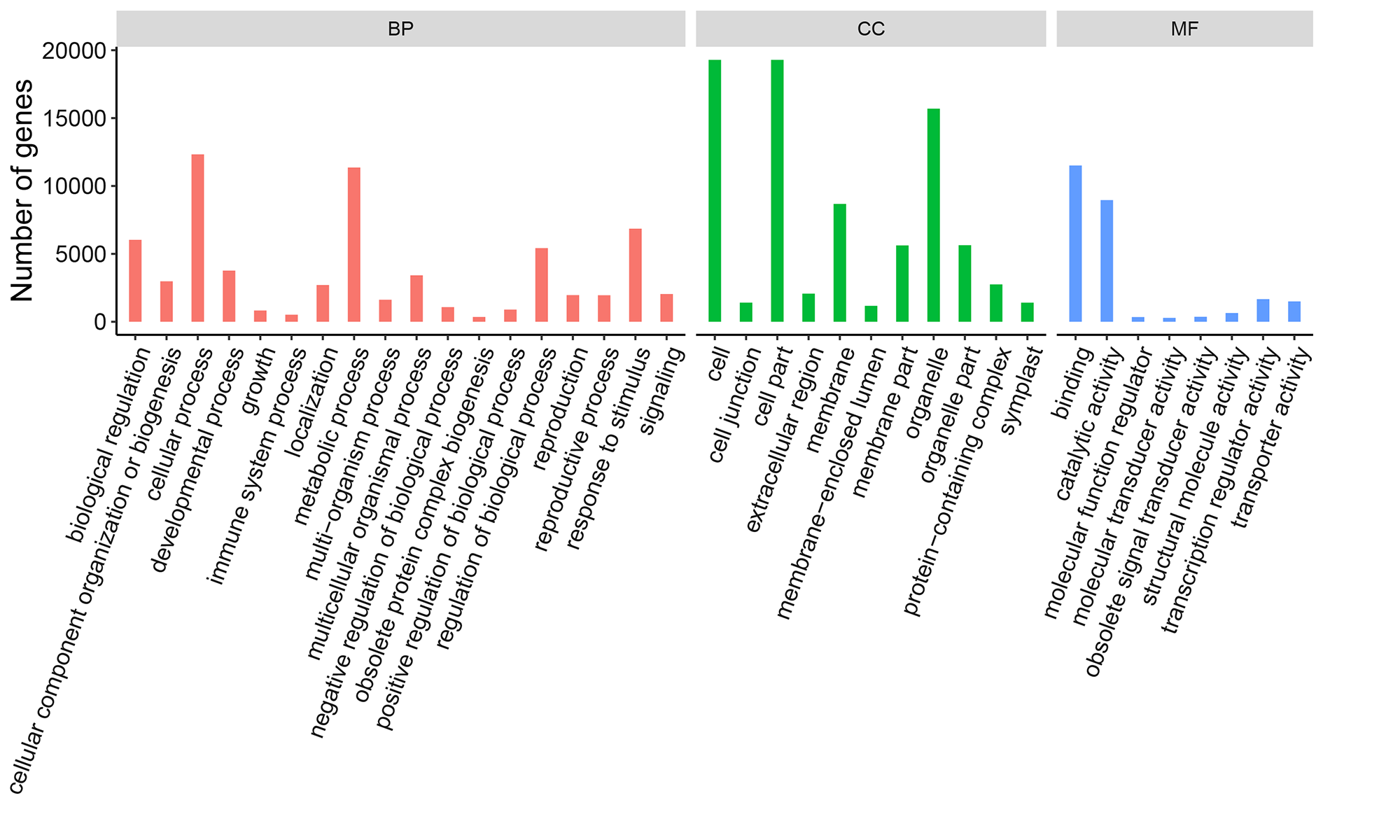

Supplement: Supplementary file 7 — Figure S7. GO term classification of G. australe genes. BP, biological process; CC, cellular component; MF, molecular function. (TIF 7061 kb) [file 12870_2019_1968_MOESM7_ESM.tif]

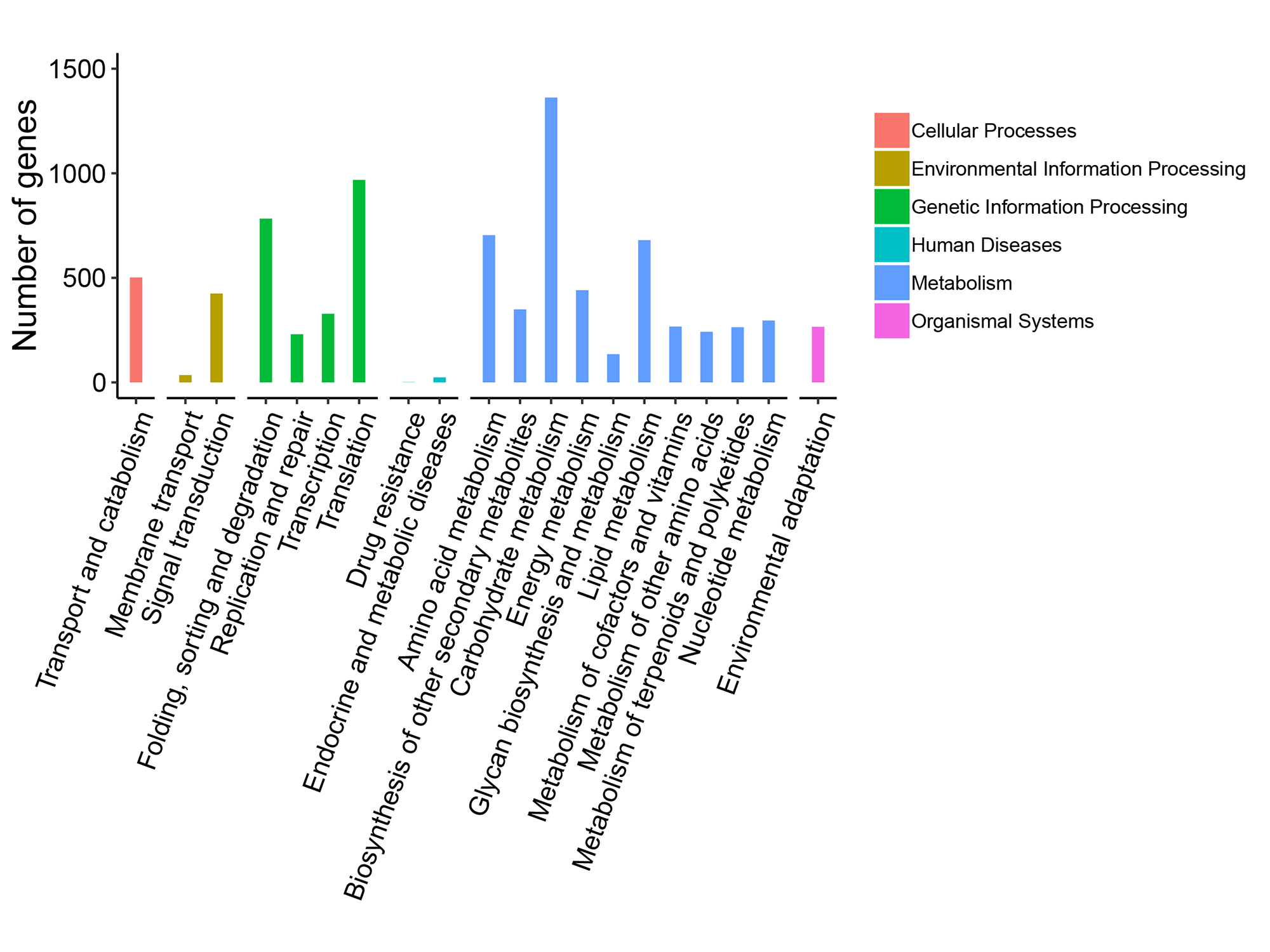

Supplement: Supplementary file 8 — Figure S8. KEGG pathway classification of G. australe genes. (TIF 8819 kb) [file 12870_2019_1968_MOESM8_ESM.tif]

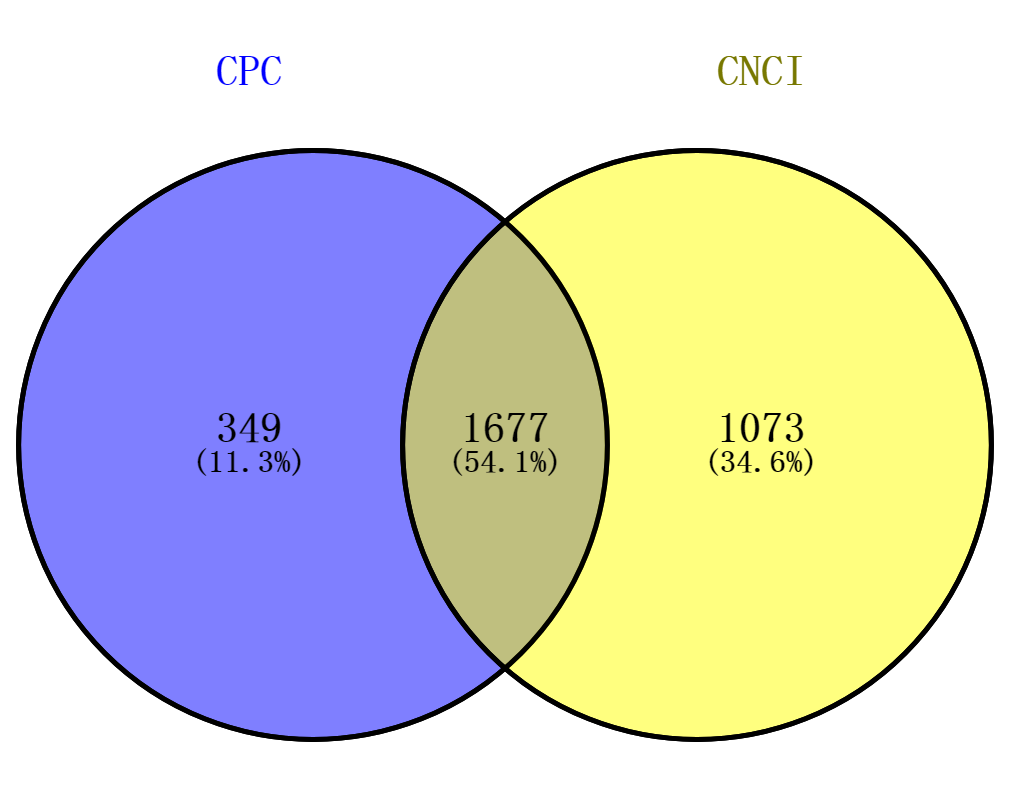

Supplement: Supplementary file 9 — Figure S9. Coding potential evaluated by CPC and CNCI. (TIF 2364 kb) [file 12870_2019_1968_MOESM9_ESM.tif]

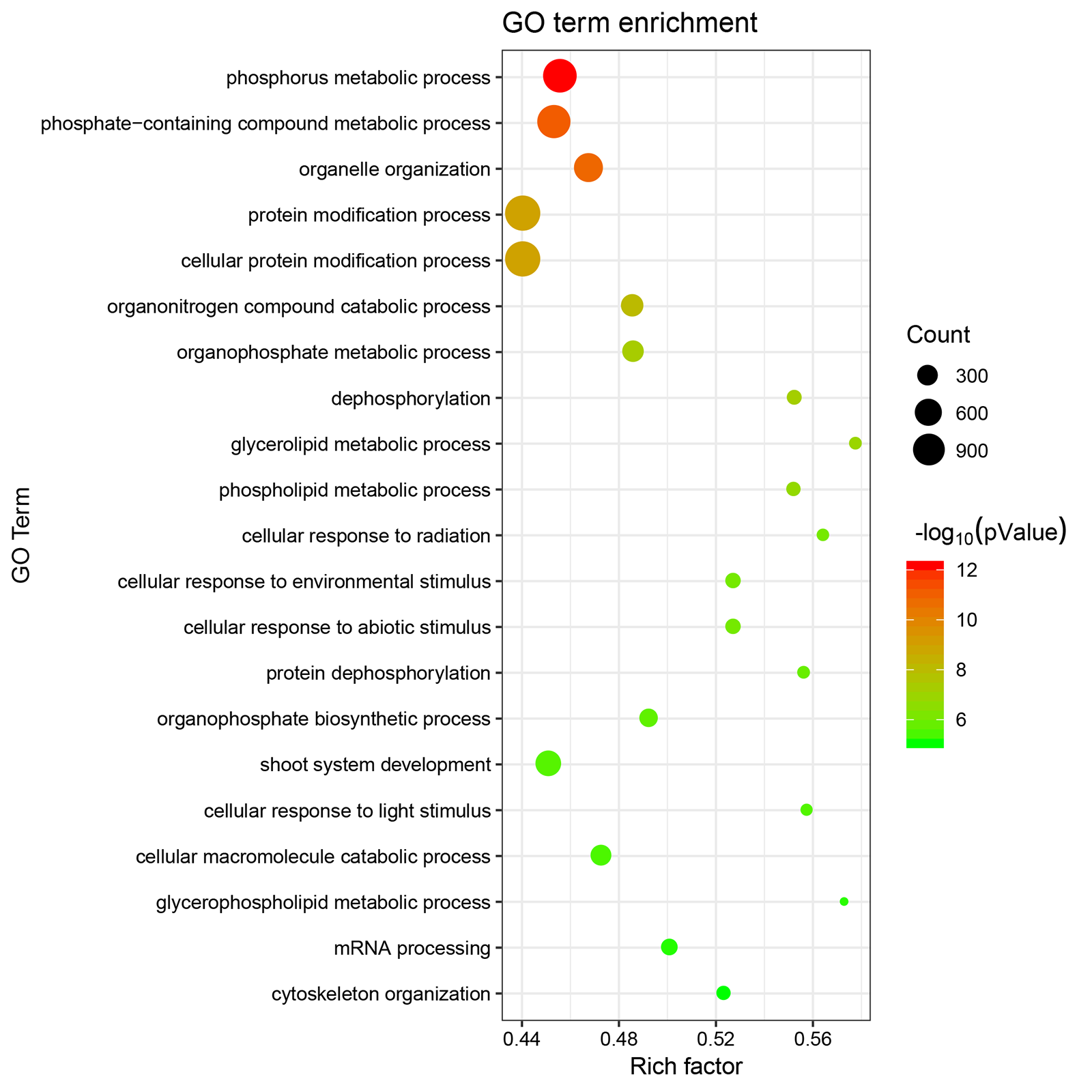

Supplement: Supplementary file 10 — Figure S10. GO enrichment analysis of AS genes. (TIF 7532 kb) [file 12870_2019_1968_MOESM10_ESM.tif]

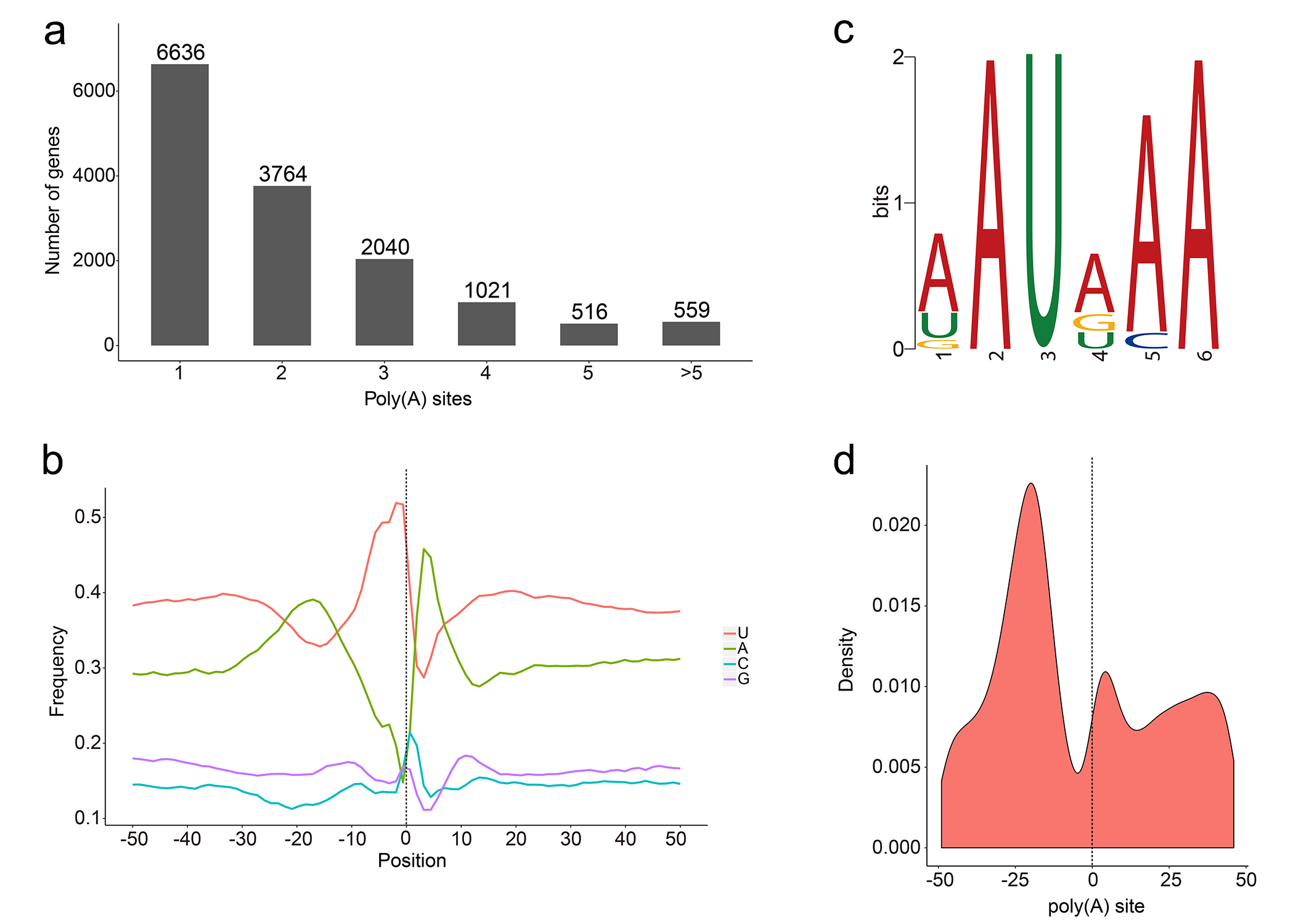

Supplement: Supplementary file 11 — Figure S11. APA analysis. a. Distribution of the number of poly(A) sites per gene. b. Nucleotide composition around poly(A) cleavage sites. c. MEME analysis of an over-represented motif at 25-nts upstream of the poly(A) site. d. Density distribution of detected motif flanking poly(A) site in our Iso-Seq data. (TIF 9733 kb) [file 12870_2019_1968_MOESM11_ESM.tif]

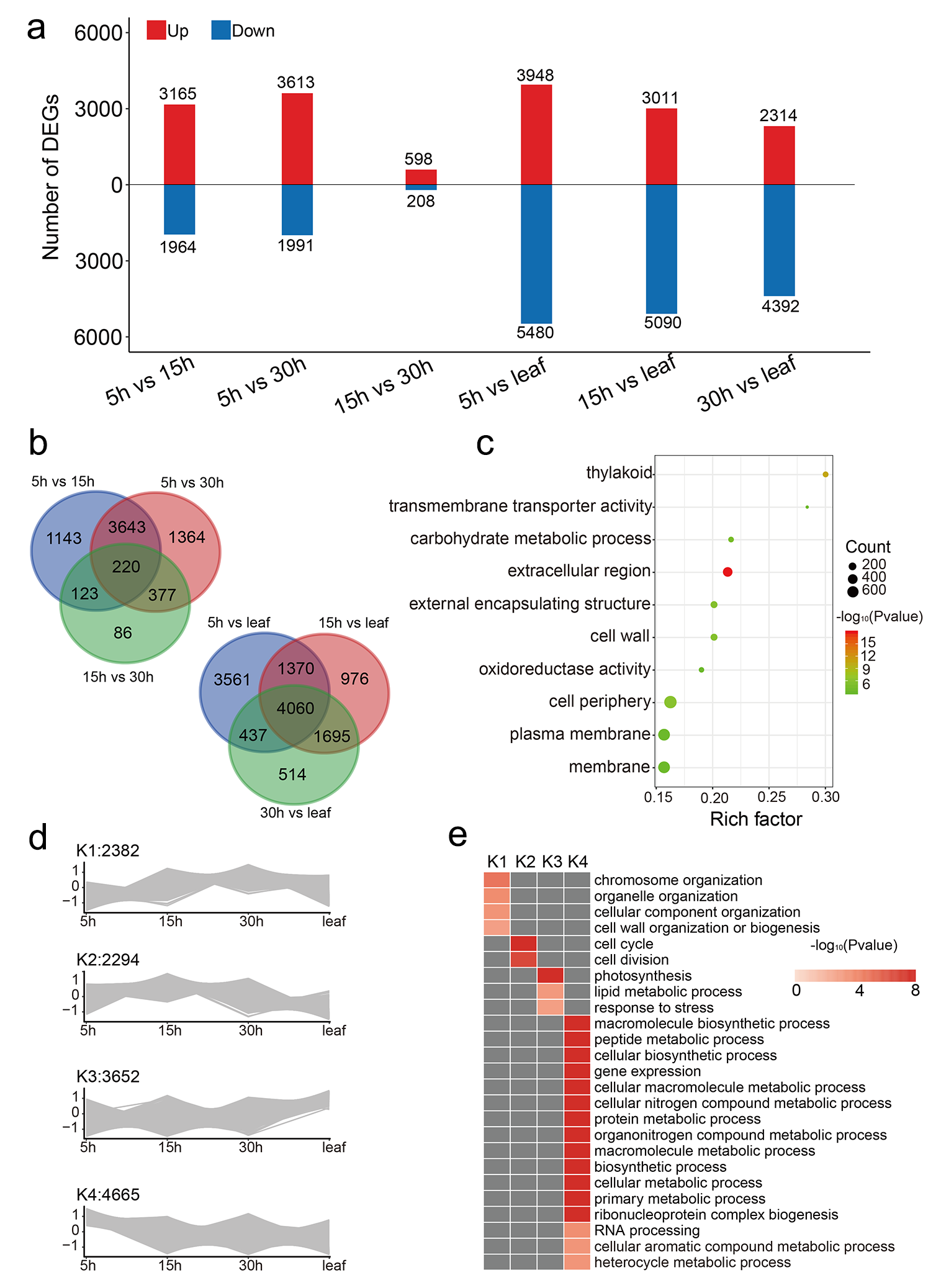

Supplement: Supplementary file 12 — Figure S12. Differentially expressed genes. a. Numbers of up- and down-regulated DEGs. b. Numbers of DEGs overlapped by pairwise comparison. c. GO enriched terms of 5 h vs 15 h and 5 h vs 30 h overlapped genes. d. Expression patterns of genes in the 4 clusters. e. Functional enrichment among the 4 clusters. (TIF 10128 kb) [file 12870_2019_1968_MOESM12_ESM.tif]

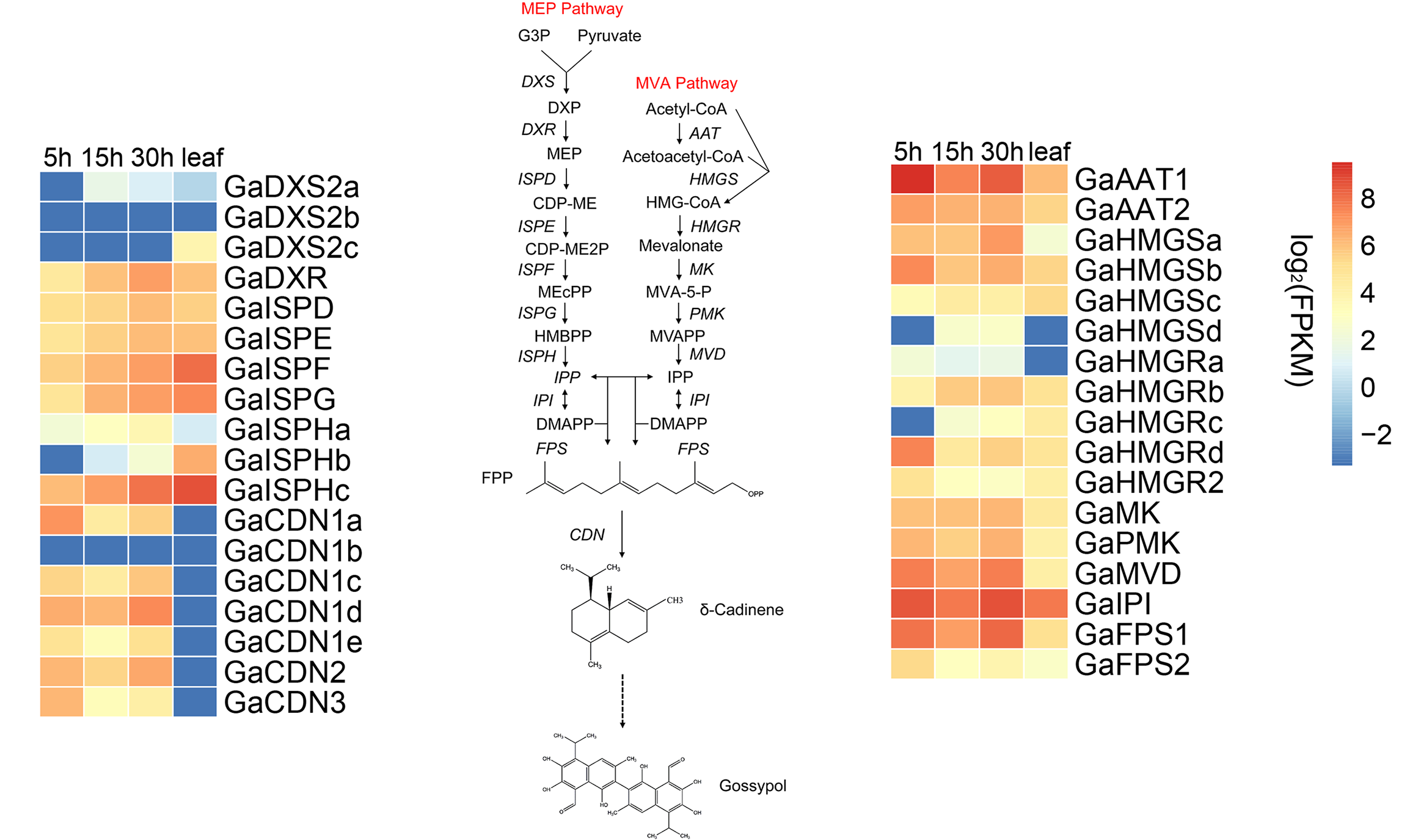

Supplement: Supplementary file 13 — Figure S13. Expression levels of genes involved in MEP- and MVA-pathway. (TIF 9397 kb) [file 12870_2019_1968_MOESM13_ESM.tif]
